# Treatment patterns and unmet needs in adults with classic congenital adrenal hyperplasia: A modified Delphi consensus study

**DOI:** 10.3389/fendo.2022.1005963

**Published:** 2022-11-18

**Authors:** Richard J. Auchus, Carine Courtillot, Adrian Dobs, Diala El-Maouche, Henrik Falhammar, Andre Lacroix, Mallory Farrar, Conor O’Donoghue, Milena Anatchkova, Katelyn Cutts, Natalie Taylor, Chuck Yonan, Mark Lamotte, Philippe Touraine

**Affiliations:** ^1^ Departments of Pharmacology and Internal Medicine, Division of Metabolism, Endocrinology and Diabetes, University of Michigan Medical School, Ann Arbor, MI, United States; ^2^ Department of Endocrinology and Reproductive Medicine, Center for Rare Endocrine and Gynecological Disorders, Groupe Hospitalier Pitié-Salpêtrière, Sorbonne Université, Paris, France; ^3^ Department of Medicine, Division of Endocrinology and Metabolism, The Johns Hopkins University School of Medicine, Baltimore, MD, United States; ^4^ Division of Endocrinology and Metabolism, George Washington University, Washington, DC, United States; ^5^ Department of Endocrinology, Karolinska University Hospital, Stockholm, Sweden; ^6^ Department of Molecular Medicine and Surgery, Karolinska Institutet, Stockholm, Sweden; ^7^ Division of Endocrinology, Department of Medicine and Research Center, Centre hospitalier de l’Université de Montréal (CHUM), Montréal, QC, Canada; ^8^ Neurocrine Biosciences, Inc., Health Economics and Outcomes Research, San Diego, CA, United States; ^9^ Neurocrine Biosciences, Inc., New Product Commercialization, San Diego, CA, United States; ^10^ Evidera, Patient-Centered Research, Bethesda, MD, United States; ^11^ IQVIA, Global Health Economics and Outcomes Research (HEOR), Zaventem, Belgium

**Keywords:** classic CAH, classic congenital adrenal hyperplasia, glucocorticoid management, treatment complication, unmet needs

## Abstract

**Background:**

Classic congenital adrenal hyperplasia (CAH) due to 21-hydroxylase deficiency is a rare autosomal recessive condition characterized by cortisol deficiency and excess androgen production. The current standard of care is glucocorticoid (GC) therapy, and sometimes mineralocorticoids, to replace endogenous cortisol deficiency; however, supraphysiologic GC doses are usually needed to reduce excess androgen production. Monitoring/titrating GC treatment remains a major challenge, and there is no agreement on assessment of treatment adequacy. This study surveyed expert opinions on current treatment practices and unmet needs in adults with classic CAH.

**Methods:**

A modified two-round Delphi process with adult endocrinologists was conducted *via* online questionnaire. Survey questions were organized into three categories: practice characteristics/CAH experience, GC management, and unmet needs/complications. Anonymized aggregate data from Round 1 were provided as feedback for Round 2. Responses from both rounds were analyzed using descriptive statistics. Consensus was defined *a priori* as: full consensus (100%, n=9/9); near consensus (78% to <100%, n=7/9 or 8/9); no consensus (<78%, n<7/9).

**Results:**

The same nine panelists participated in both survey rounds; five (56%) were based in North America and four (44%) in Europe. Most panelists (78%) used hydrocortisone in the majority of patients, but two (22%) preferred prednisone/prednisolone. Panelists agreed (89%) that adequate control is best evaluated using a balance of clinical presentation and androgen/precursor laboratory values; no consensus was reached on optimal timing of collecting samples for androgen testing or laboratory values indicating good control. Despite lack of consensus on many aspects of CAH management, panelists agreed on the importance of many disease- and GC-related complications, and that there is a large unmet need for new treatments. With currently available treatments, panelists reported that 46% of classic CAH patients did not have optimized androgen levels, regardless of GC dose.

**Conclusions:**

The limited areas of consensus obtained in this study reflect the variability in treatment practices for adults with classic CAH, even among clinicians with expertise in treating this population. However, all panelists agreed on the need for new treatments for classic CAH and the importance of many disease- and GC-related complications, which are difficult to manage with currently available treatments.

## 1 Introduction

Congenital adrenal hyperplasia (CAH) refers to a group of rare autosomal recessive disorders that result in disordered adrenal steroidogenesis, including impaired cortisol and aldosterone synthesis (1–5). Approximately 95-99% of CAH cases are the result of mutations in the *CYP21A2* gene encoding for the adrenal steroidogenic enzyme, 21-hydroxylase, which is required for the production of cortisol and aldosterone in the adrenal cortex (1, 6). Severe blockage of cortisol synthesis reduces normal negative feedback inhibition on the hypothalamus and the pituitary gland, leading to increased secretion of adrenocorticotropic hormone (ACTH) and excess production of adrenal androgens and their precursors (1–5).

The “classic” form of CAH is associated with severe 21-hydroxylase deficiency and occurs in approximately 1:14,000 to 1:18,000 births (1). High intrauterine androgen concentrations are clinically evident in newborn females, whose external genitalia are virilized to varying degrees, while males with classic CAH usually have typical male genitalia (7, 8).

Androgen excess during childhood and adolescence raises the risk for precocious puberty or pseudopuberty, as well as accelerated somatic growth with advanced bone age, which results in below-predicted adult height (2, 7). During adulthood, females can develop hirsutism, acne, and irregular menses; males are at risk of developing testicular adrenal rest tumors (TARTs). Both males and females are at risk for long-term problems with bone health, cardiovascular and metabolic comorbidities, fertility, and psychosocial health and well-being, due to the disease and/or its conventional treatments (2, 7, 9–11). Patients of all ages are at risk of adrenal gland nodular enlargement and adrenal crisis, which is potentially life-threatening if untreated (2, 7, 9, 12).

Management of classic CAH presents unique challenges distinct from other forms of adrenal insufficiency (1–5, 8). The current standard of care is glucocorticoid (GC) therapy, with or without mineralocorticoid treatment, to replace the endogenous cortisol deficiency and reduce excess androgen production. However, unlike acquired primary adrenal insufficiency, supraphysiologic GC doses are usually needed to simultaneously reduce the elevated ACTH secretion and excess androgen production (3, 4). Chronic exposure to supraphysiologic GC doses can lead to a number of potentially serious health complications, including growth suppression and decreased bone density with increased fracture risk, as well as metabolic complications such as obesity, insulin resistance, hypertension, and diabetes, which can increase cardiovascular risk (9, 13–20). Thus, the need for adequate androgen control should be balanced with the risks of prolonged supraphysiologic GC exposure, as both under- and overtreatment with GCs can cause complications. In addition to these challenges, there is a lack of consensus among practitioners on optimal GC regimens in adult patients (4, 8, 21). Although hydrocortisone in divided doses is a common treatment option for adults, once- or twice-daily preparations of synthetic long-acting GCs such as prednisone, prednisolone, and dexamethasone are also used; modified-release hydrocortisone has recently gained approval from the European Medicines Agency (1, 3, 4, 22, 23). In addition, monitoring and titrating GC treatments remains a major clinical challenge, and there is no agreement on the assessment of treatment adequacy (1, 4, 8).

The purpose of this study was to survey expert opinions on current GC treatment practices and unmet needs in adult patients with classic CAH. The Delphi method, a systematic group communication process, was developed by the RAND Corporation in the 1950s to forecast the impact of technology on warfare and is well suited to assist in decision-making when evidence is incomplete, unclear, or unavailable (24–28). The iterative and anonymous nature of the traditional Delphi questioning process, with analysis and feedback provided after survey rounds, represents a structured process to collect knowledge from a panel of experts, with the capability of achieving consensus when uncertainty may exist due to lack of definitive evidence (24–28). Panelists’ anonymity during the survey process can reduce the effects of dominant individuals or pressure to conform, which often is a concern when using group-based processes to collect and synthesize information. Controlled feedback in the form of a well-organized summary of the prior iteration allows each participant an opportunity to generate additional insights, clarify the information developed in previous iterations, and minimize the effects of noise. The Delphi method has been used successfully for various medical applications, from the assessment of knowledge gaps to the development of treatment guidelines (29–33). This study utilizes a modified Delphi method to survey expert opinions on the management of adult patients with classic CAH, as well as unmet needs in this patient population.

## 2 Methods

### 2.1 Expert panel

**Figure 1:**
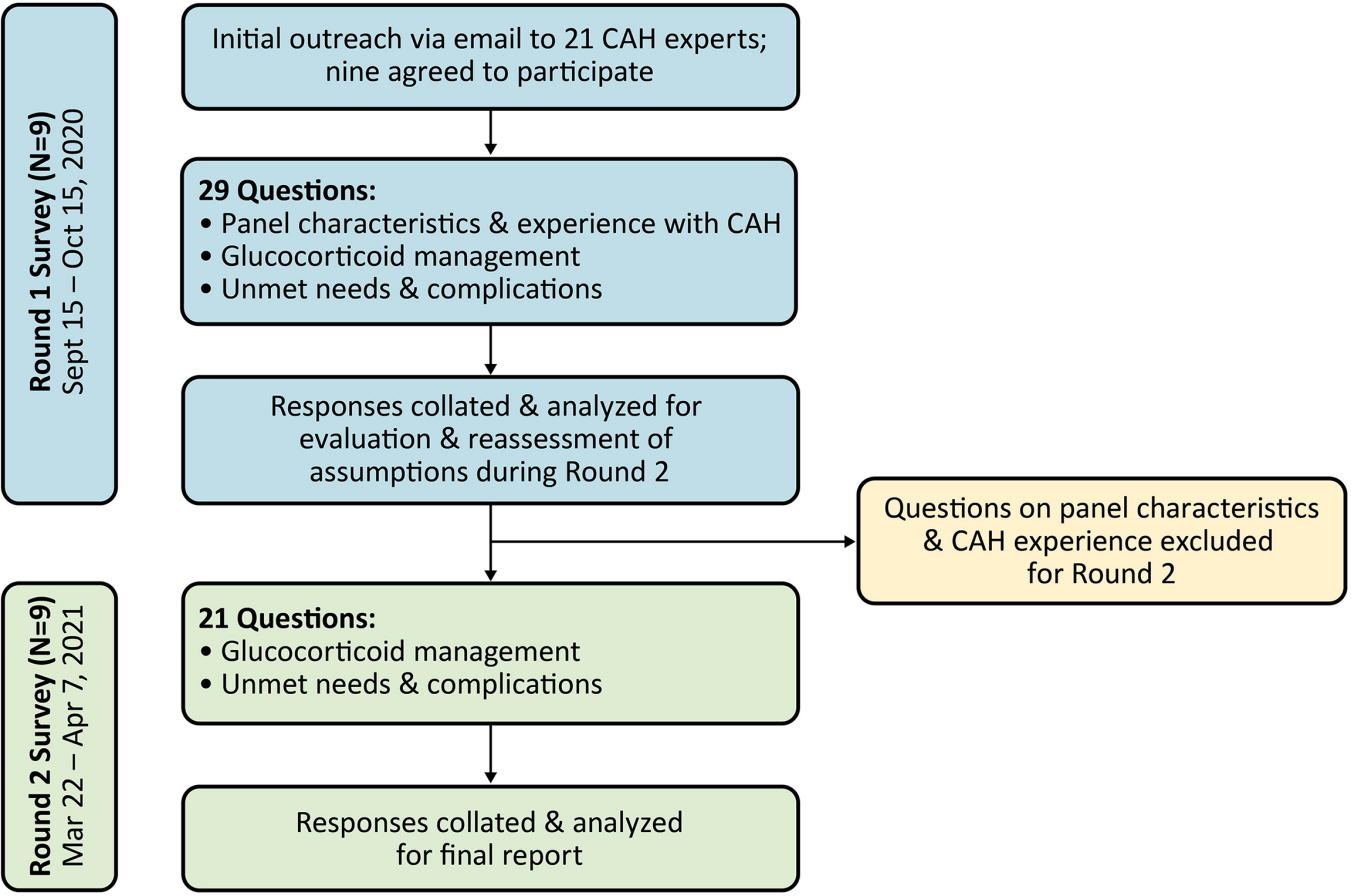
Flowchart of modified Delphi panel process. CAH, congenital adrenal hyperplasia.

Survey panelists from the US, Canada, and Europe were recruited by Evidera upon recommendation by Neurocrine Biosciences, Inc., the study sponsor. Participation was voluntary, but respondents were compensated for their time in completing the survey. Recruitment efforts for the survey panel focused on adult endocrinologists who frequently managed patients with classic CAH (i.e., currently seeing at least 10-20 adults with classic CAH every quarter). Additional criteria for recruitment included involvement in publications on CAH, participation in CAH clinical trials, or participation in the development of CAH guidelines. Of the 21 panelists invited to participate in the survey panel, nine agreed to participate (**Figure 1**). All nine panelists completed both rounds of the survey, and seven participated in the development of this paper (two panelists did not participate in manuscript preparation and have elected to remain anonymous).

### 2.2 Modified Delphi procedure

Two internet-based survey rounds were conducted from September 2020 to April 2021 (**Figure 1**). The questions were organized into three categories: 1) panel members’ practice characteristics and CAH experience (Round 1 only), 2) GC management (including GC treatment patterns, hydrocortisone equivalency ratios, target androgen laboratory values and indicators of good control, and treatment optimization), and 3) unmet needs and complications.

The Delphi process traditionally begins with an open-ended (free-response) questionnaire in Round 1, but a common and acceptable modification is to use more structured questions if basic information on the target issue is available (27). In this study, most Round 1 questions were free-response, but some closed-ended questions (e.g., 5-point Likert scale [select rating of 1 “not important” to 5 “very important”] or multiple choice [select 1 or select any]) were used based on input from several prior virtual Advisory Boards with CAH experts on current treatment practices and unmet needs, as well as analysis by Neurocrine to identify potential gaps in research, published literature, and treatment guidelines for classic CAH (survey questionnaires are shown in **Supplementary Materials**). Responses were collected and collated by Evidera and analyzed by Evidera, Neurocrine, and IQVIA.

For Round 2, panelists were provided anonymous aggregate data from Round 1 (and their individual responses from Round 1 where applicable) as feedback. Questions for Round 2 were refined as follows: 1) If there was general agreement in Round 1 responses, panelists were asked if they agreed or disagreed with the conclusion to establish consensus; 2) If there was variability in Round 1 responses, the question was re-circulated (and in some cases, modified for clarity) for a second round of input from panelists.

### 2.3 Analysis

Quantitative responses from both survey rounds were analyzed using descriptive statistics, including mean, standard deviation, range, and frequency. Qualitative data from the free-response questions were analyzed using key codes developed with clinical input from Neurocrine. For Round 2 survey results, consensus was defined *a priori* as follows: full consensus (100%, n=9/9); near consensus (78% to <100%, n=8/9 or 7/9); no consensus (<78%, n<7/9).

## 3 Results

### 3.1 Panel characteristics and experience with CAH

Of the nine total panel members, five (56%) were based in North America, and four (44%) were based in Europe (**Supplementary Table 1**). All nine panelists were adult endocrinologists, with the majority working in an academic or university hospital setting. Most of the panelists had ≥15 years of experience treating adults with CAH and were currently treating ≥10 adult patients with classic CAH. In an average month, the panel members reported spending a median of 4% of their time treating adults with classic CAH.

### 3.2 Glucocorticoid management

Survey results on GC treatment patterns, hydrocortisone equivalency ratios, target androgen laboratory values, and treatment optimization are presented in the following sections. Key findings are summarized in **Box 1**.

**Box 1**

Key Findings: Glucocorticoid Management in Adults with Classic CAH. ✔ indicates full consensus (100%, 9/9 respondents); **✔** indicates near consensus (78% to <100%, 8/9 or 7/9 respondents); **✘** indicates no consensus (<78%, <7/9 respondents). 17-OHP, 17-hydroxyprogesterone; A4, androstenedione; CAH, congenital adrenal hyperplasia; GC, glucocorticoid; ULN, upper limit of normal.

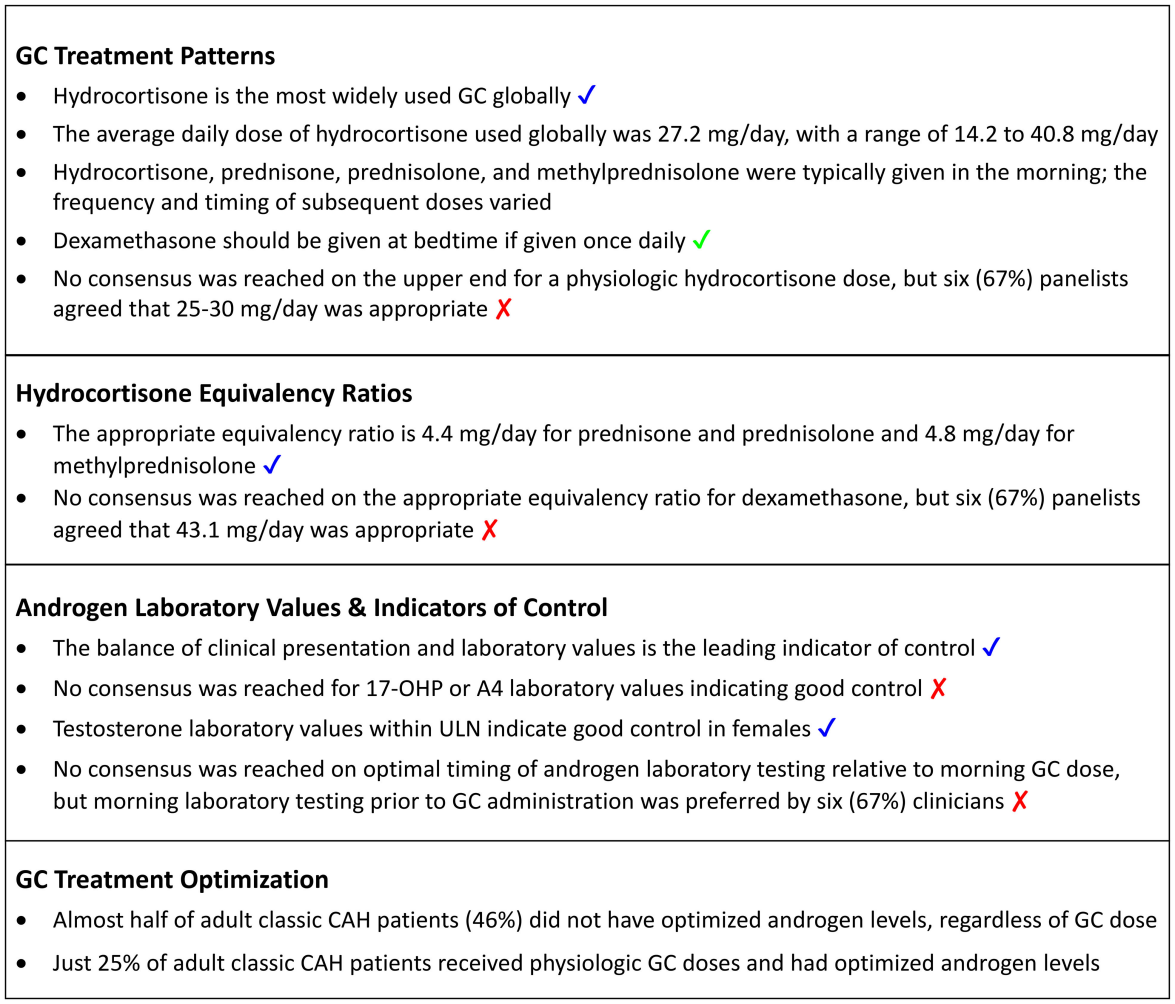

#### 3.2.1 Glucocorticoid treatment patterns

In Round 1, all nine panelists reported prescribing hydrocortisone in an average of 62% of patients, with individual responses ranging from 10% to 96% of patients (**Table 1**). Most panelists (78%) prescribed hydrocortisone in more than half of their patients; the other two (22%) panelists used prednisone or prednisolone in 80% of patients. Six panelists reported prescribing dexamethasone in an average of 8% of their patients. When asked to select up to three different GC combinations they typically used in their practice, a total of five panelists reported using at least one GC combination: three used only hydrocortisone and dexamethasone; one used hydrocortisone with dexamethasone, prednisone, or prednisolone; and one used hydrocortisone with prednisolone or methylprednisolone.

**Table 1:** Glucocorticoid treatment patterns in adults with classic CAH.

	Hydrocortisone	Dexamethasone	Prednisone	Prednisolone	Methylprednisolone
**% of patients taking GC**					
Number of respondents who used GC in >0% of patients	9	6	5	3	1
Mean (SD)	62 (33)	8 (7)	26 (32)	32 (42)	25
Median (range)	65 (10-96)	3 (2-20)	10 (5-80)	10 (5-80)	25
**Daily GC dose, mg/d** ^a^					
Number of respondents	8	7	6	5	5
Mean (SD)	27.2 (3.6)	0.6 (0.3)	4.9 (1.5)	4.4 (0.9)	5.4 (1.3)
Median (range)	25.0 (25.0-35.0)	0.5 (0.4-1.0)	5.0 (3.0-7.5)	5.0 (3.0-5.0)	6.0 (3.0-6.0)
**Daily GC dose range, mg/d** ^a^					
Number of respondents	6	5	5	4	4
Lower range, mean (SD)	14.2 (3.8)	0.4 (0.1)	3.8 (1.8)	3.3 (1.7)	3.8 (2.1)
Upper range, mean (SD)	40.8 (10.2)	1.5 (0.6)	7.5 (1.8)	7.1 (2.2)	6.5 (1.9)
**Timing of GC dosing, n (%) respondents**					
Number of respondents	9	6	5	3	1
Morning	9 (100)	1 (17)	4 (80)	3 (100)	1 (100)
Afternoon	8 (89)	0 (0)	1 (20)	1 (33)	0 (0)
Evening	7 (78)	1 (17)	0 (0)	1 (33)	0 (0)
Bedtime	2 (22)	5 (83)	3 (60)	1 (33)	1 (100)
Uses reverse circadian dosing	1 (11)	2 (33)	1 (20)	0 (0)	0 (0)

a Some panelists provided both a number and a range for the average daily GC dose.

GC, glucocorticoid; SD, standard deviation.

When asked in Round 2 to provide the average daily GC dose and/or average dose range used globally to treat patients with classic CAH, the mean hydrocortisone dose was 27.2 mg/day, and the mean hydrocortisone dose range was 14.2 to 40.8 mg/day (**Table 1**). For dexamethasone, the panelists reported an average daily dose of 0.6 mg/day and a range of 0.4 to 1.5 mg/day. When asked about the typical timing of GC doses, nine (100%) panelists prescribed the first dose of hydrocortisone in the morning; 89% also prescribed it in the afternoon, 78% in the evening, and 22% at bedtime (**Table 1**). The timing and frequency of dosing for prednisone, prednisolone, or methylprednisolone varied among panelists, but the first dose was usually in the morning. Dexamethasone was usually dosed at bedtime. Two (33%) panelists reported using reverse circadian dosing.

In Round 2, near consensus was reached that hydrocortisone is the most widely used GC globally (89%), and that dexamethasone should be prescribed at bedtime if given once daily (78%) (**Box 1**).

#### 3.2.2 Physiologic hydrocortisone dose

When asked in Round 1 to provide what they considered to be the upper end of a physiologic GC dose with hydrocortisone, panelists reported a mean dose of 27.2 mg/day, with individual responses ranging from 15 to 40 mg/day – which was consistent with hydrocortisone dosing that the panelists reported using in clinical practice (see previous section). All but one panelist felt that the dose they indicated was reliable (some risk of being wrong, seven [78%]) or certain (low risk of being wrong, one [11%]). In Round 2, consensus agreement was not reached, but six (67%) panelists agreed that 25-30 mg/day was the upper end for a physiologic hydrocortisone dose (**Box 1**). The three panelists who disagreed with this dose range provided doses of 15, 20, and 40 mg/day.

#### 3.2.3 Hydrocortisone equivalency ratios

In Round 1, panelists were asked to provide an appropriate hydrocortisone equivalency ratio to use when summarizing a data set (i.e., not in clinical practice, but when reading a peer-reviewed article). The mean (SD, range) hydrocortisone equivalency ratios reported by panelists were as follows: dexamethasone, 43.1 (25.4, 25.0-80.0); prednisone, 4.4 (0.5, 4.0-5.0); prednisolone, 4.4 (0.5, 4.0-5.0); methylprednisolone, 4.8 (0.4, 4.0-5.0). In Round 2, all but one panelist (89%) agreed that the Round 1 equivalency ratios for prednisone, prednisolone, and methylprednisolone were appropriate to use when summarizing a dataset (**Box 1**). Consensus was not reached for dexamethasone, but six (67%) panelists agreed with the appropriateness of the Round 1 equivalency ratio (**Box 1**). The three panelists who disagreed reported that the equivalency ratio for dexamethasone should be 25.0, 26.7, or 80.0 mg/day.

#### 3.2.4 Androgen laboratory values and indicators of control

**Figure 2:**
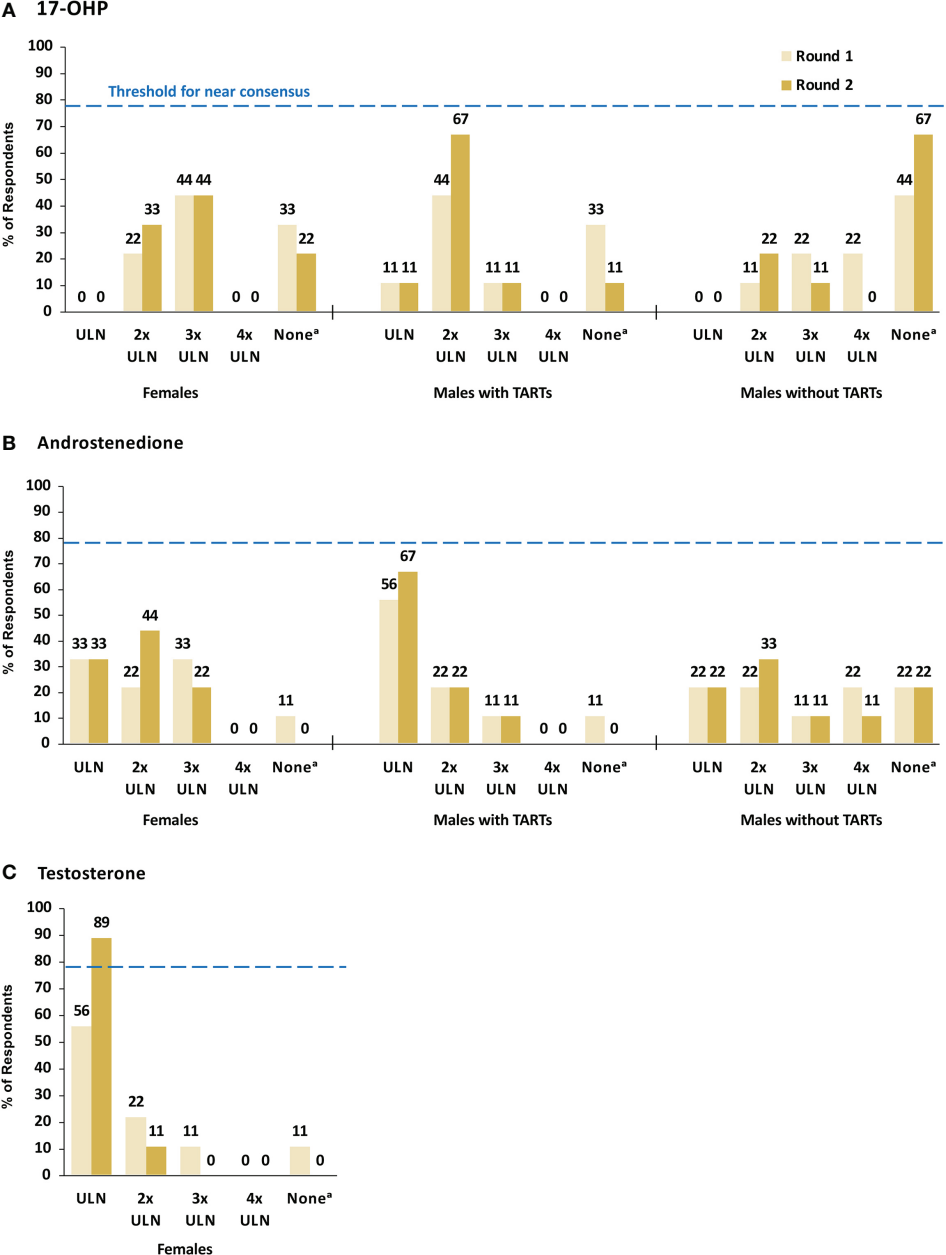
17-OHP **(A)**, Androstenedione **(B)**, and Testosterone **(C)** Laboratory Values Indicating Good Control. Dashed line indicates minimum threshold for near consensus (78%, or 7/9 respondents). ^a^ The survey response option for this category was “I do not have a lab range I treat to”. 17-OHP, 17-hydroxyprogesterone; TARTs, testicular adrenal rest tumors; ULN, upper limit of normal.

**Figure 2** summarizes responses to questions in Round 1 and Round 2 on what laboratory values for 17-hydroxyprogesterone (17-OHP), androstenedione (A4), and testosterone are considered appropriate indicators of good control in three patient subgroups: males with TARTs, males without TARTs, and females. For 17-OHP, consensus was not reached in any subgroup on an appropriate laboratory value to indicate good control. In males with TARTs, a total of six (67%) panelists agreed in Round 2 that within 2X the upper limit of normal (ULN) was appropriate, but this did not meet the threshold for near consensus (**Figure 2A** and **Box 1**). Of the three panelists who responded in Round 1 that they did not treat to a specific 17-OHP range, two panelists modified their response in Round 2 to 2X ULN. For males without TARTs, six (67%) panelists agreed in Round 2 that they did not have a 17-OHP laboratory range that they treated to. For females, most panelists reported that within 2X ULN (33%) or 3X ULN (44%) was appropriate for 17-OHP.

For A4, consensus was not reached in any subgroup on an appropriate laboratory value to indicate good control, but six (67%) panelists agreed in Round 2 that within ULN was appropriate in males with TARTs (**Figure 2B** and **Box 1**). There was no agreement on an appropriate A4 laboratory value in males without TARTs. For females, most panelists reported that within ULN (33%) or 2X ULN (44%) was appropriate for A4. For testosterone, near consensus (89%) was reached in Round 2 that within ULN was an appropriate testosterone laboratory value to indicate good control in females (**Figure 2C** and **Box 1**).

In Round 2, near consensus (89%) was reached that the leading indicator of control is the balance of clinical presentation and laboratory values (**Box 1**). Based on comments received in Round 1 that the expectations for appropriate target laboratory values would vary depending on the timing of the laboratory testing relative to the administration of the GC dose, a question was added to Round 2 to indicate the optimal timing of laboratory testing. Consensus was not reached by the panelists, but morning laboratory testing prior to GC administration was preferred by six (67%) panelists (**Box 1**).

#### 3.2.5 Glucocorticoid treatment optimization

**Figure 3:**
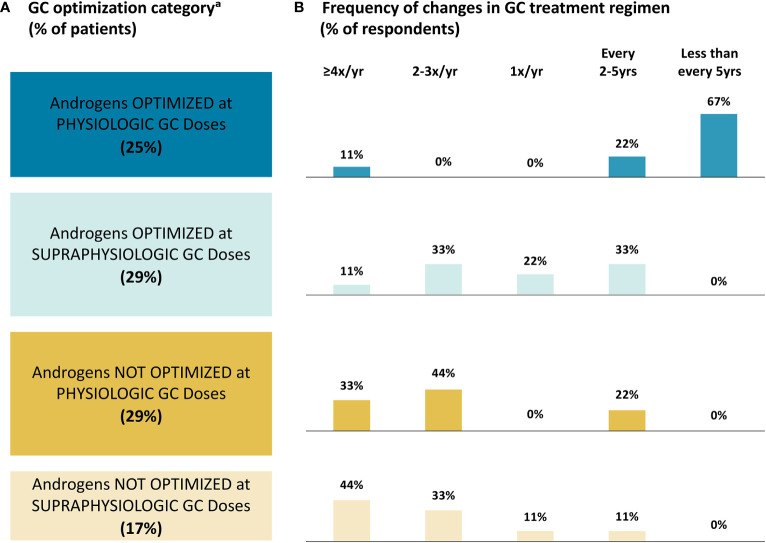
Glucocorticoid Treatment Optimization. **(A)** Patient Category; **(B)** Frequency of Changes. ^a^ The survey questionnaires did not define “optimized” androgen levels or “physiologic” GC doses; therefore, panelists reported percentages of patients in each category based on their own definitions of optimized androgens and physiologic GCs. GC, glucocorticoid; yr, year.

Based on their own definitions for what they considered to be “optimized” androgen levels and “physiologic” GCs, panelists reported that almost half of their adult patients with classic CAH (46%) did not have optimized androgen levels, regardless of GC doses (**Figure 3A**). A total of 29% of patients had androgens optimized but were receiving supraphysiologic GC doses. Just 25% of patients received physiologic GC doses and had optimized androgen levels.

Panelists reported the most frequent GC treatment regimen changes in patients whose androgens were not optimized, with seven (78%) panelists reporting making changes at least twice a year in these patients (**Figure 3B**). There was a lack of agreement on the frequency of changes for patients whose androgens are optimized with supraphysiologic GC doses, with four (44%) panelists reporting changes at least twice a year and five (56%) panelists reporting changes every one to five years. Panelists reported the least frequent changes in patients with androgens optimized and physiologic GC doses, with eight (89%) panelists reporting changes every two to five or more years. When asked to provide reasons for changing a patient’s GC regimen (free-response question), six (67%) panelists reported factors related to good androgen control/hyperandrogenism, with five (56%) panelists specifically mentioning fertility. A total of four (44%) panelists reported factors related to managing supraphysiologic GC doses.

### 3.3 Exploring unmet needs and complications in adults with classic CAH

Survey results on unmet needs and the relative importance of disease- and GC-related complications are presented in the following sections, and key findings are summarized in **Box 2**.

**Box 2**

Key Findings: Unmet Needs and Complications in Adults with Classic CAH. ✔ indicates full consensus (100%, 9/9 respondents); **✔** indicates near consensus (78% to <100%, 8/9 or 7/9 respondents); **✘** indicates no consensus (<78%, <7/9 respondents). CAH, congenital adrenal hyperplasia; GC, glucocorticoid; TARTs, testicular adrenal rest tumors.

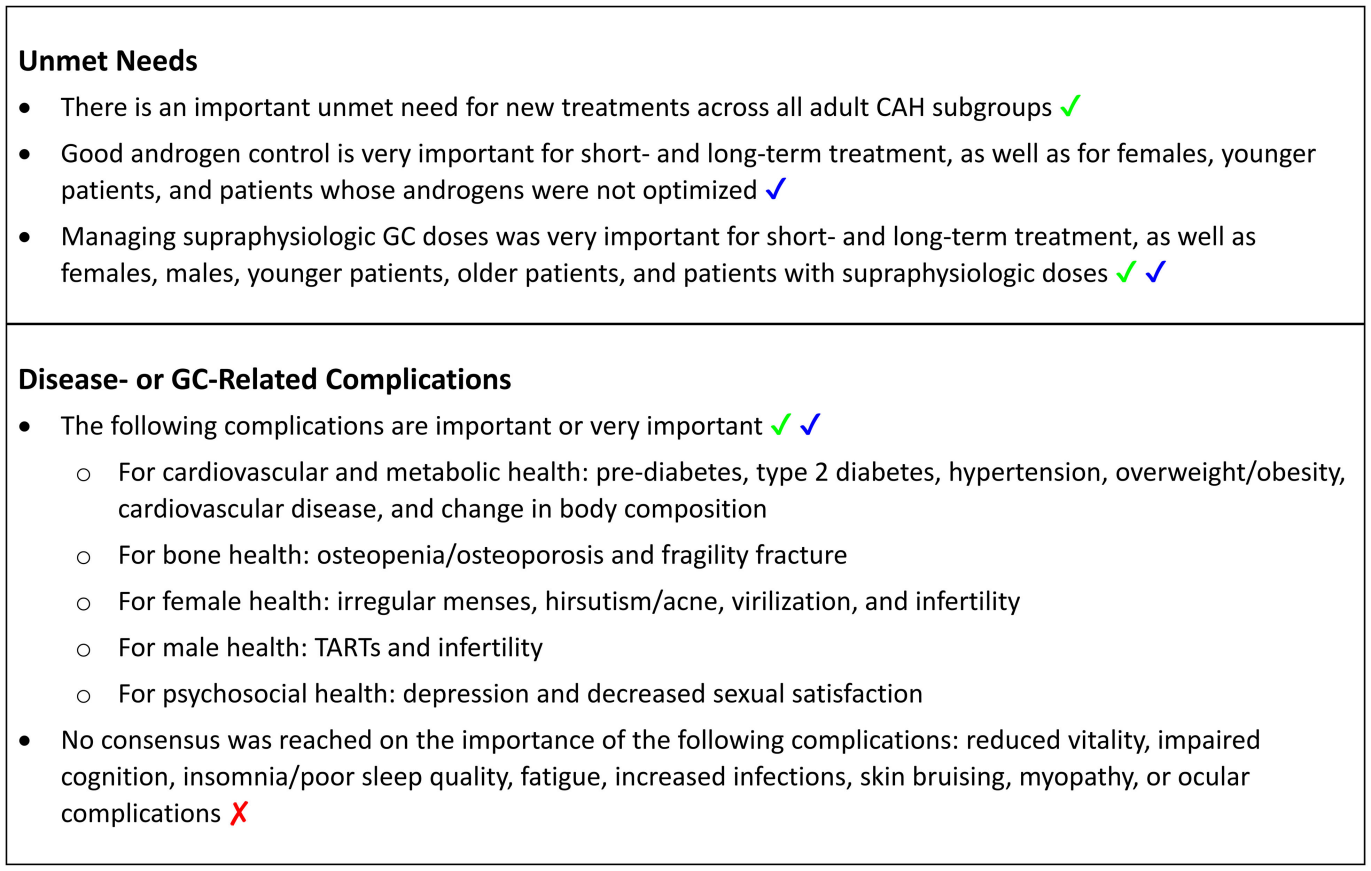

#### 3.3.1 Unmet needs

In Round 1, the panelists were asked free-response questions to provide the most important short- and long-term unmet needs, as well as the most important unmet needs for classic CAH patients categorized by sex, age, and GC treatment optimization. Based on responses in Round 1, the unmet needs were categorized into three groups for Round 2 questioning:

o Good androgen control/avoidance of hyperandrogenism (including fertility, androgen replacement therapy, and hirsutism)
o Managing/reducing supraphysiologic GC doses (including consequences related to cardiovascular, bone, and metabolic health)
o Treatment-related needs (including simplified dosing and affordability)

In Round 2, the panel ranked the importance of the three unmet needs categories from 1 “not at all important” to 5 “very important”. Near consensus (78%) was reached that good androgen control was very important for short- and long-term treatment, as well as for females, younger patients (age 18 to ≤55 years), and patients whose androgens were not optimized (**Supplementary Table 2** and **Box 2**). Consensus or near consensus was reached that managing supraphysiologic GC doses was very important for short- and long-term, females, males, younger patients, older patients (age >55 years), and patients with supraphysiologic GC doses. Consensus (100%) was also reached that there is an important unmet need for new treatments across all adult CAH subgroups (**Box 2**).

#### 3.3.2 Disease- and GC-related complications

In Round 1, panelists were asked to rank the importance of several disease- or GC-related complications from 1 “not at all important” to 5 “very important”. Based on the mean response from Round 1, panelists were asked in Round 2 if they agreed (yes/no) that the complication was “important/very important” (mean ≥4, Round 1) or “moderately important” (mean <4, Round 1). In Round 2, consensus (100%) or near consensus (78%) was reached that all of the complications related to cardiovascular and metabolic health, bone health, female health, and male health were important or very important, except dyslipidemia, which the panel agreed was moderately important (78%) (**Table 2** and **Box 2**). No consensus was reached for most of the complications related to psychosocial health and well-being, except for depression and decreased sexual satisfaction, which panelists agreed were important or very important, and anxiety, which panelists agreed was moderately important. No consensus was reached on “other” complications, such as increased infections, skin bruising, myopathy, and ocular complications.

**Table 2:** Importance of disease- or GC-related complications in adults with classic CAH.

	Round 1					Round 2	
Complication, n (%) of respondents	Not at all important	Somewhat important	Moderately important	Important	Very Important	Do you agree that the complication is important/very important or moderately important?	
						Yes	No
**Cardiovascular and metabolic health**						**Important or Very Important?**	
Pre-diabetes	0 (0)	1 (11)	1 (11)	4 (44)	3 (33)	**9 (100)**	0 (0)
Type 2 diabetes	0 (0)	2 (22)	1 (11)	1 (11)	5 (56)	**9 (100)**	0 (0)
Hypertension	0 (0)	2 (22)	0 (0)	3 (33)	4 (44)	**9 (100)**	0 (0)
Overweight/obesity	0 (0)	0 (0)	0 (0)	4 (44)	5 (56)	**9 (100)**	0 (0)
Cardiovascular disease	0 (0)	1 (11)	0 (0)	2 (22)	6 (67)	**9 (100)**	0 (0)
Other: change in body composition	0 (0)	0 (0)	0 (0)	1 (100)^a^	0 (0)	**7 (78)**	2 (22)
						**Moderately Important?**	
Dyslipidemia	0 (0)	1 (11)	2 (22)	3 (33)	3 (33)	**7 (78)**	2 (22)
**Bone health**						**Important or Very Important?**	
Osteopenia/osteoporosis	0 (0)	0 (0)	1 (11)	4 (44)	4 (44)	**9 (100)**	0 (0)
Fragility fracture	0 (0)	0 (0)	1 (11)	2 (22)	6 (67)	**9 (100)**	0 (0)
**Female health**						**Important or Very Important?**	
Irregular menses/ anovulation/amenorrhea	0 (0)	0 (0)	1 (11)	6 (67)	2 (22)	**9 (100)**	0 (0)
Hirsutism/acne	0 (0)	0 (0)	1 (11)	6 (67)	2 (22)	**9 (100)**	0 (0)
Virilization	1 (11)	0 (0)	0 (0)	2 (22)	6 (67)	**9 (100)**	0 (0)
Infertility	0 (0)	0 (0)	0 (0)	2 (22)	**7 (78)**	Not asked (consensus reached in R1)	
**Male health**						**Important or Very Important?**	
TARTs	0 (0)	0 (0)	0 (0)	3 (33)	6 (67)	**9 (100)**	0 (0)
Infertility	0 (0)	0 (0)	0 (0)	2 (22)	**7 (78)**	Not asked (consensus reached in R1)	
**Psychosocial health and well-being**						**Important or Very Important?**	
Depression	0 (0)	1 (11)	0 (0)	5 (56)	3 (33)	**9 (100)**	0 (0)
Decreased sexual satisfaction	0 (0)	1 (11)	0 (0)	6 (67)	2 (22)	**9 (100)**	0 (0)
						**Moderately Important?**	
Anxiety	0 (0)	1 (11)	1 (11)	5 (56)	2 (22)	**7 (78)**	2 (22)
Reduced vitality	0 (0)	0 (0)	4 (44)	3 (33)	2 (22)	5 (56)	4 (44)
Impaired cognition	0 (0)	3 (33)	1 (11)	2 (22)	3 (33)	3 (33)	6 (67)
Insomnia/poor sleep quality	0 (0)	2 (22)	2 (22)	3 (33)	2 (22)	5 (56)	4 (44)
Fatigue	0 (0)	1 (11)	3 (33)	3 (33)	2 (22)	5 (56)	4 (44)
**Other complications**						**Moderately Important?**	
Increased infections	0 (0)	2 (22)	2 (22)	3 (33)	2 (22)	6 (67)	3 (33)
Skin bruising/thinning/ fragility	0 (0)	1 (11)	2 (22)	4 (44)	2 (22)	6 (67)	3 (33)
Myopathy	0 (0)	1 (11)	2 (22)	3 (33)	3 (33)	6 (67)	3 (33)
Ocular (glaucoma, cataracts)	0 (0)	1 (11)	4 (44)	2 (22)	2 (22)	6 (67)	3 (33)

Green indicates full consensus (100%, 9/9 respondents), blue indicates near consensus (78% to <100%, 8/9 or 7/9 respondents), and red indicates no consensus (<78%, <7/9 respondents).

a One respondent listed “change in body composition” under “Other” in Round 1.

R1, round 1; TARTs, testicular adrenal rest tumors.

## 4 Discussion

Despite the advances in the past several decades in the understanding of the genetics, pathophysiology, and treatment of classic CAH, many challenges remain in managing the condition (1, 4, 5, 8, 21). The lifelong supraphysiologic GC doses that are often needed to attenuate the excess adrenal-derived androgen production are associated with a high burden of comorbidities and reduced quality of life in adult patients with classic CAH (9, 17–20). Thus, clinicians must balance the need for adequate androgen control with the risks of health problems from prolonged supraphysiologic GC exposure. Adding to this challenge is the limited evidence from randomized trial data comparing long-term outcomes of different GCs and GC regimens in adults, leading to a lack of consensus on how to optimize GC therapy (1, 4, 34).

This study aimed to provide a view of expert opinions on current practices and unmet needs in the management of adult patients with classic CAH. The survey results showed some areas of agreement in GC management, including near consensus that hydrocortisone was the most widely used GC. Most panelists reported using hydrocortisone in the majority of their patients, but two panelists preferred the long-acting GCs, prednisone or prednisolone. These findings align with recent published reviews, which describe hydrocortisone split in two to three doses as the most common treatment option for adult patients due to its lower risk of adverse effects on metabolic, cardiovascular, and bone health (1, 3, 7). Long-acting synthetic GCs were often used for regulation of menstrual cycles, fertility induction, TART treatment, or patients who have difficulty adhering to a three-times daily regimen, but their longer duration and higher potency may increase the risk of metabolic comorbidities (1, 7).

A potential limitation of this study is the small number of panel participants, whose opinions might not reflect those of other endocrinologists who treat adults with CAH. In addition, the small number of panelists meant that consensus or near consensus would not be reached if only one or three panelists gave dissenting opinions, respectively, which could skew results. To ensure a representative sample of expert opinions, panelists from different institutional and clinical settings throughout the US and Europe were recruited who met academic and clinical criteria. However, classic CAH is a rare disorder; thus, there were few clinicians who met the study inclusion criteria of seeing at least 10-20 adults with classic CAH every quarter. Larger studies surveying a broader geographical range of expert opinions (beyond the US and Europe) may expand our findings and help to provide a more comprehensive, global view of adult CAH care. Financial support for the study was provided by Neurocrine, who is investigating crinecerfont, a corticotropin-releasing factor type 1 receptor (CRF1R) antagonist, for potential use in CAH. To mitigate potential bias introduced by the commercial sponsor, the survey questions were specifically designed to address a broad and comprehensive clinical approach to the management of classic CAH.

Typical daily GC doses reported by the panelists in this study generally aligned with the dose ranges suggested in the 2018 Endocrine Society guidelines and in recent literature, although the panelists reported higher upper ranges for hydrocortisone (40.8 vs 25 mg) and dexamethasone (1.5 vs 0.5 mg) (1, 3, 7). These findings are in alignment with cross-sectional studies of adults with classic CAH in the UK (20) and the US (35), which found a wide variation of GC regimens among clinical practice settings in both countries. A recent retrospective study of children with classic CAH in the International-CAH registry (www.i-cah.org) also revealed large variations in GC treatments and doses (36).

There was a lack of consensus among panelists on what they considered to be a physiologic hydrocortisone dose for adults, but the majority agreed that 25-30 mg was the appropriate upper end for a physiologic hydrocortisone dose range. This lack of consensus is reflected in published estimates of physiologic hydrocortisone dose, which ranged from 7.5-15 mg/m^2^/day, or approximately 15-25 mg/day of hydrocortisone (37, 38). However, prior studies in children with classic CAH have shown that a hydrocortisone dose of 8 mg/m^2^/day was not associated with clinical manifestations of GC insufficiency, and these data suggest that 8 mg/m^2^/day (or approximately 15 mg/day in adults) might be an adequate physiologic dose (39, 40).

In terms of the timing of GC dose administration, consensus was reached that once-daily dexamethasone should be administered at bedtime. The panelists typically prescribed hydrocortisone three times daily, starting in the morning. There was less agreement on the timing and frequency of dosing for prednisone, prednisolone, and methylprednisolone, but the first dose was usually given in the morning.

When asked about best practices for patient monitoring, panelists agreed that adequate control is best evaluated using the balance of clinical presentation and androgen/precursor laboratory values, but there was a lack of consensus on optimal timing for androgen/precursor laboratory testing and 17-OHP and A4 laboratory values indicating good control. The Endocrine Society recommends monitoring treatment through annual physical examinations and consistently timed biochemical measurements to assess the adequacy of GC treatment; however, the guidelines do not include specific recommendations on how to time the measurements or what the target levels should be (3). More recently, it has been suggested that the use of biomarkers such as 21-deoxycortisol and 11-oxysteriods may provide more direct evidence of adrenal androgen precursor production and thereby improve monitoring and titrating of current GC regimens; however, the use of these biomarkers has not been established in clinical care (1, 3). In addition, Saevik et al. recently proposed the use of circulating mRNA from GC-responsive genes, such as *DSIPI*, *DDIT4*, and *FKBP5*, as potential biomarkers in patients with autoimmune Addison’s disease; however, further research is needed to explore the potential and validity of transcriptional biomarkers for GC replacement therapy (41).

The lack of agreement among panelists in most areas of GC management reflects the difficulties in using a “population” level approach for treating patients with classic CAH and suggests the need for a patient-specific approach in this population. A “treat-to-target” approach, as used in diabetes and dyslipidemia, is generally not appropriate; rather, treatment decisions should include careful consideration of the individual characteristics of each patient, including age, gender, genetic background (e.g., GC receptor polymorphisms), treatment goals, and side effects to guide shared decision making. This need for individualized treatment is also reflected in the relatively broad Endocrine Society treatment guidelines, which recommend the use of daily hydrocortisone and/or long-acting GCs plus mineralocorticoids for adults with classic CAH “as clinically indicated”, with limited guidance on treatment optimization or patient monitoring (3).

Despite the lack of consensus on many aspects of CAH management, there was consensus agreement on the importance of many disease- and GC-related complications. In addition, all panelists agreed that there is a large unmet need for new treatments. With the currently available treatment options, panelists reported that almost half of their patients with classic CAH did not have optimized androgen levels, and another 29% had androgens optimized but were receiving supraphysiologic GC doses. Just 25% of patients were receiving physiologic GC doses and were perceived to have optimized androgen levels. These findings are in agreement with the previously mentioned cross-sectional studies in the UK and US, in which only 36% and 40% of adults with classic CAH, respectively, had normal serum A4 levels (20, 35).

Newer therapies, such as modified release hydrocortisone preparations and alternative hydrocortisone delivery systems (continuous subcutaneous infusion), have been developed as alternatives to long-acting synthetic GCs (42–44). Studies of these therapies indicated improved biomarker control, but GC exposure remained >20 mg/day (42–44). Bilateral adrenalectomy has been attempted as a strategy for management of classic CAH with lower (physiologic) GC dosing similar to the approach used for acquired primary adrenal insufficiency, but this approach is associated with a risk of short- and long-term adverse outcomes, including development of adrenal rest tumors (even in women) and an increased risk of adrenal crisis (45, 46). A promising strategy is the development of adjunctive therapies to reduce androgen production without the need for supraphysiologic GC dosing. Abiraterone acetate for six days added to 20 mg/day hydrocortisone normalized A4 in six adult women with classic CAH (47), but longer studies have not been performed. Crinecerfont, a CRF1R antagonist, was shown in a phase 2 trial to lower ACTH and afford clinically meaningful reductions of elevated 17-OHP, A4, testosterone (women), or A4/testosterone ratio (men) (48). Phase 3 trials of crinecerfont are currently ongoing. Another CRF1R antagonist, tildacerfont, was shown in 14-day and three-month phase 2 trials to reduce ACTH, 17-OHP and A4 levels (testosterone levels were not reported in this study) (49). These potential treatments and others are discussed in more detail elsewhere (1, 2).

## 5 Conclusions

The limited areas of consensus obtained in this study reflect the variability in treatment practices for adults with classic CAH, even among clinicians with expertise in treating this population. The management of classic CAH is heterogeneous and varies widely by patient and provider; there is no single agreed-upon way to treat or manage classic CAH. However, this study found full consensus on the need for new treatments for classic CAH and the importance of many disease- and GC-related complications, which are difficult to manage with currently available therapeutic options.

## Data availability statement

The original contributions presented in the study are included in the article/**Supplementary Material**, Further inquiries can be directed to the corresponding author.

## Author contributions

MF, CO’D, CY, and ML contributed to the conception and design of the Delphi methodology. RA, CC, AD, DE-M, HF, AL, and PT were expert panel members and survey participants. MA, KC, and NT collected and collated the survey responses. MA, KC, NT, and ML contributed to the analysis and interpretation of the results. All authors contributed to the drafting and critical revision of this manuscript and approved the submitted version.

## Funding

This study was supported by Neurocrine Biosciences, Inc., San Diego, CA.

## Acknowledgments

Survey responses were collected and collated by Evidera (Bethesda, MD) and analyzed by Evidera and IQVIA (Zaventem, Belgium), with support from Neurocrine Biosciences, Inc. (San Diego, CA). Medical writing and editorial services were provided by Prescott Medical Communications Group (Chicago, IL), with support from Neurocrine.

## Conflict of interest

MF, CO’D and CY were employed by Neurocrine Biosciences, Inc. MA, KC, and NT were employed by Evidera. ML, full-time employee of IQVIA. IQVIA received consulting fees for the advice of ML on this research. RA received research funding from Neurocrine Biosciences, Inc., Diurnal, LTD, and Spruce Biosciences; RA served as consultant for Neurocrine Biosciences, Inc., Crinetics Pharmaceuticals, OMass Therapeutics, H Lundbeck A/S, and Adrenas Therapeutics. HF served as consultant for Neurocrine Biosciences, Inc., Diurnal Ltd., Roche Diagnostics International Ltd., H Lundbeck A/S, and Adrenas Therapeutics. AL served as editor Adrenal Section, UpToDate.

The authors declare that this study received funding from Neurocrine Biosciences, Inc. The funder had the following involvement in the study: Contracted with Evidera and IQVIA to conduct the Delphi surveys, collate data, perform statistical analyses, and assist with interpretation of data and preparation of the manuscript. Contracted with Prescott Medical Communications Group to assist with preparation of the manuscript.

## Publisher’s note

All claims expressed in this article are solely those of the authors and do not necessarily represent those of their affiliated organizations, or those of the publisher, the editors and the reviewers. Any product that may be evaluated in this article, or claim that may be made by its manufacturer, is not guaranteed or endorsed by the publisher.
