# Supplementary material for: Treatment patterns and unmet needs in adults with classic congenital adrenal hyperplasia: A modified Delphi consensus study
Source: Front Endocrinol (Lausanne). 2022 Nov 18;13:1005963. doi: 10.3389/fendo.2022.1005963 (PMC9717438; doi:10.3389/fendo.2022.1005963)
Supplement: Supplementary file 1 [file DataSheet_1.docx]

**SUPPLEMENTARY MATERIALS**

**Supplementary Table 1. Panel Characteristics and Experience with CAH**

| **Characteristic, n (%)** | **Delphi Panel Respondents**  **N=9** |
| --- | --- |
| **Practice location** |  |
| United States | 4 (44) |
| France | 2 (22)^a^ |
| Canada | 1 (11) |
| Sweden | 1 (11) |
| United Kingdom | 1 (11) |
| **Practice setting** |  |
| Academic/university hospital | 6 (67) |
| Specialized CAH center | 3 (33) |
| Private practice | 0 (0) |
| **Endocrinology specialty** |  |
| Adult | 9 (100) |
| Pediatric | 0 (0) |
| Both (adult and pediatric) | 0 (0) |
| **Experience treating adult patients with classic CAH, years** |  |
| <5 | 1 (11) |
| 5-15 | 0 (0) |
| 15-20 | 4 (44) |
| >20 | 4 (44) |
| **Current adult CAH patients (classic and non-classic)** |  |
| <10 | 0 (0) |
| 10-25 | 3 (33) |
| 25-50 | 3 (33) |
| >50 | 3 (33) |
| **Current adult CAH patients (classic)** |  |
| <5 | 0 (0) |
| 5-10 | 1 (11) |
| 10-20 | 4 (44) |
| >20 | 4 (44) |

^a^ The 2 panelists from France were part of the same university hospital practice.

CAH, congenital adrenal hyperplasia.

**Supplementary Table 2. Unmet Needs in Adults with Classic CAH**

| **Unmet Need, n (% Respondents)^a^** | **Round 1** | **Round 2** | | | | |
| --- | --- | --- | --- | --- | --- | --- |
|  |  | **Not at all Important** | **Somewhat Important** | **Moderately Important** | **Important** | **Very Important** |
| Short-term (all patients) |  |  |  |  |  |  |
| Good androgen control/hyperandrogenism | 5 (56) | 0 (0) | 0 (0) | 0 (0) | 2 (22) | **7 (78)** |
| Managing/reducing supraphysiologic GC doses | 5 (56) | 0 (0) | 0 (0) | 0 (0) | 0 (0) | **9 (100)** |
| Treatment-related needs | 4 (44) | 0 (0) | 0 (0) | 1 (11) | 4 (44) | 4 (44) |
| Long-term (all patients) |  |  |  |  |  |  |
| Good androgen control/hyperandrogenism | 5 (56) | 0 (0) | 0 (0) | 0 (0) | 2 (22) | **7 (78)** |
| Managing/reducing supraphysiologic GC doses | 6 (67) | 0 (0) | 0 (0) | 0 (0) | 0 (0) | **9 (100)** |
| Treatment-related needs | 2 (22) | 0 (0) | 1 (11) | 0 (0) | 5 (56) | 3 (33) |
| Females |  |  |  |  |  |  |
| Good androgen control/hyperandrogenism | 8 (89) | 0 (0) | 0 (0) | 0 (0) | 2 (22) | **7 (78)** |
| Managing/reducing supraphysiologic GC doses | 2 (22) | 0 (0) | 0 (0) | 0 (0) | 0 (0) | **9 (100)** |
| Treatment-related needs | 1 (11) | 0 (0) | 1 (11) | 1 (11) | 5 (56) | 2 (22) |
| Males |  |  |  |  |  |  |
| Good androgen control/hyperandrogenism | 8 (89) | 0 (0) | 0 (0) | 1 (11) | 2 (22) | 6 (67) |
| Managing/reducing supraphysiologic GC doses | 2 (22) | 0 (0) | 0 (0) | 0 (0) | 1 (11) | **8 (89)** |
| Treatment-related needs | 1 (11) | 0 (0) | 1 (11) | 1 (11) | 5 (56) | 2 (22) |
| Younger (18 to ≤55 years) |  |  |  |  |  |  |
| Good androgen control/hyperandrogenism | 7 (78) | 0 (0) | 0 (0) | 0 (0) | 2 (22) | **7 (78)** |
| Managing/reducing supraphysiologic GC doses | 5 (56) | 0 (0) | 0 (0) | 0 (0) | 2 (22) | **7 (78)** |
| Treatment-related needs | 1 (11) | 0 (0) | 1 (11) | 1 (11) | 5 (55) | 2 (22) |
| Older (>55 years) |  |  |  |  |  |  |
| Good androgen control/hyperandrogenism | 0 (0) | - | | | | |
| Managing/reducing supraphysiologic GC doses | 7 (78) | 0 (0) | 0 (0) | 0 (0) | 2 (22) | **7 (78)** |
| Treatment-related needs | 0 (0) | - | | | | |
| Other: adrenal enlargement | 1 (11) | 0 (0) | 4 (44) | 5 (56) | 0 (0) | 0 (0) |
| Androgens optimized, physiologic GC doses | | | | | | |
| Good androgen control/hyperandrogenism | 0 (0) | - | | | | |
| Managing/reducing supraphysiologic GC doses | 1 (11) | 4 (44) | 2 (22) | 1 (11) | 2 (22) | 0 (0) |
| Treatment-related needs | 2 (22) | 0 (0) | 4 (44) | 2 (22) | 2 (22) | 1 (11) |
| There is no unmet need | 3 (33) | 3 (33) | 2 (22) | 2 (22) | 1 (11) | 1 (11) |
| Other: adrenal crisis, psychological support, long-term maintenance | 3 (33) | 0 (0) | 4 (44) | 1 (11) | 0 (0) | 4 (44) |
| Androgens optimized, supraphysiologic GC doses | | | | | | |
| Good androgen control/hyperandrogenism | 1 (11) | 0 (0) | 3 (33) | 2 (22) | 2 (22) | 2 (22) |
| Managing/reducing supraphysiologic GC doses | 8 (89) | 0 (0) | 0 (0) | 1 (11) | 0 (0) | **8 (89)** |
| Treatment-related needs | 1 (11) | 0 (0) | 3 (33) | 1 (11) | 0 (0) | 5 (56) |
| Androgens NOT optimized, physiologic GC doses | | | | | | |
| Good androgen control/hyperandrogenism | 8 (89) | 0 (0) | 0 (0) | 0 (0) | 2 (22) | **7 (78)** |
| Managing/reducing supraphysiologic GC doses | 0 (0) | - | | | | |
| Treatment-related needs | 0 (0) | - | | | | |
| Other: adrenal enlargement | 1 (11) | 1 (11) | 4 (44) | 4 (44) | 0 (0) | 0 (0) |
| Androgens NOT optimized, supraphysiologic GC doses | | | | | | |
| Good androgen control/hyperandrogenism | 5 (56) | 0 (0) | 0 (0) | 1 (11) | 1 (11) | **7 (78)** |
| Managing/reducing supraphysiologic GC doses | 6 (67) | 0 (0) | 0 (0) | 0 (0) | 0 (0) | **9 (100)** |
| Treatment-related needs | 1 (11) | 0 (0) | 4 (44) | 0 (0) | 2 (22) | 3 (33) |

**Green** indicates full consensus (100%, 9/9 respondents), **blue** indicates near consensus (78 to <100%, 8/9 or 7/9 respondents), and **red** indicates no consensus (<78%, <7/9 respondents).

^a^ Good androgen control/hyperandrogenism included improving fertility, regaining regular periods, exploring androgen replacement therapy, and reducing hirsutism; managing/reducing supraphysiologic GC doses included preventing consequences related to cardiovascular, bone, and metabolic health; treatment-related needs included simplified dosing to enhance compliance, more affordable treatment options, and hormone replacement or mineralocorticoid therapies that control androgens without side effects.

CAH, congenital adrenal hyperplasia; GC, glucocorticoid.

**Round 1 Delphi Questionnaire**

**Part 1. Panel Member Demographics and Experience with Congenital Adrenal Hyperplasia.** *First, please answer a few questions about your clinical background.*

1. What is your specialty?

□ Adult endocrinology

□ Pediatric endocrinology

□ Both adult and pediatric endocrinology

□ Other: ___________

1. What is the clinical setting(s) where you primarily see classic CAH patients?

□ Specialized CAH Center

□ Academia or University Hospital

□ Private practice

□ Other: ___________

1. How many adult (18 years or older) patients with **CAH** (**classic or non-classic**) do you manage/follow in your practice currently?

□ Less than 10 patients

□ 10-25 patients

□ 25-50 patients

□ More than 50 patients

1. How many adult patients with **classic CAH** do you manage/follow in your practice currently?

□ Less than 5 patients

□ 5-10 patients

□ 10-20 patients

□ More than 20 patients

1. In an average month, what % of your time seeing patients is with adult patients with **CAH** (**classic and non-classic**)?
   _______%
2. In an average month, what % of your time seeing patients is with adult patients with **classic CAH**?
   _______%
3. How many years have you worked in a clinical setting seeing adult patients with **classic CAH**?

□ Less than 5 years

□ 5-10 years

□ 10-15 years

□ 15-20 years

□ More than 20 years

1. Where is your practice located?

Country: ___________________

City: ___________________

**Part 2. Glucocorticoid Management**. *Please answer the following questions based on your experience managing adult (18 years or older) patients with classic CAH.*

1. In your practice, ROUGHLY what percentage of adult patients with classic CAH are on each of the following glucocorticoid (GC) regimens, at present? [Answers should sum to 100 %]

Hydrocortisone only ______ %

Dexamethasone only ______ %

Prednisone only ______ %

Prednisolone only ______ %

Methylprednisolone only ______ %

Combination of above ______ %

Other: ____________ ______ %

Sum: ______ %

Please select the glucocorticoids you typically use in combination.

Combination 1 Please select your answer ____

Combination 2 Please select your answer ____

Combination 3 Please select your answer ____

1. What is the total daily dose you **typically** use for the treatment of adult patients with classic CAH for each of the glucocorticoid regimens below? *Please enter a number or a range.*

Number OR Range

Hydrocortisone ______ mg/day ______ - ______ mg/day

Dexamethasone ______ mg/day ______ - ______ mg/day

Prednisone ______ mg/day ______ - ______ mg/day

Prednisolone ______ mg/day ______ - ______ mg/day

Methylprednisolone ______ mg/day ______ - ______ mg/day

Combination medication #1: Hydrocortisone ______ mg/day ______ - ______ mg/day

Combination medication #1: Dexamethasone ______ mg/day ______ - ______ mg/day

Combination medication #1: Prednisone ______ mg/day ______ - ______ mg/day

Combination medication #1: Prednisolone ______ mg/day ______ - ______ mg/day

Combination medication #1: Methylprednisolone ______ mg/day ______ - ______ mg/day

Combination medication #2: Hydrocortisone ______ mg/day ______ - ______ mg/day

Combination medication #2: Dexamethasone ______ mg/day ______ - ______ mg/day

Combination medication #2: Prednisone ______ mg/day ______ - ______ mg/day

Combination medication #2: Prednisolone ______ mg/day ______ - ______ mg/day

Combination medication #2: Methylprednisolone ______ mg/day ______ - ______ mg/day

Combination medication #3: Hydrocortisone ______ mg/day ______ - ______ mg/day

Combination medication #3: Dexamethasone ______ mg/day ______ - ______ mg/day

Combination medication #3: Prednisone ______ mg/day ______ - ______ mg/day

Combination medication #3: Prednisolone ______ mg/day ______ - ______ mg/day

Combination medication #3: Methylprednisolone ______ mg/day ______ - ______ mg/day

1. For each of the below glucocorticoids, what is the typical time you prescribe glucocorticoid administration and do you use reverse circadian dosing? (check all that apply)

| Glucocorticoid | Morning | Afternoon | Evening | Bedtime | Reverse circadian dosing?  Check if yes |
| --- | --- | --- | --- | --- | --- |
| Hydrocortisone | □ | □ | □ | □ | □ |
| Dexamethasone | □ | □ | □ | □ | □ |
| Prednisone | □ | □ | □ | □ | □ |
| Prednisolone | □ | □ | □ | □ | □ |
| Methylprednisolone | □ | □ | □ | □ | □ |
| Combination medication #1: Hydrocortisone | □ | □ | □ | □ | □ |
| Combination medication #1: Dexamethasone | □ | □ | □ | □ | □ |
| Combination medication #1: Prednisone | □ | □ | □ | □ | □ |
| Combination medication #1: Prednisolone | □ | □ | □ | □ | □ |
| Combination medication #1: Methylprednisolone | □ | □ | □ | □ | □ |
| Combination medication #2: Hydrocortisone | □ | □ | □ | □ | □ |
| Combination medication #2: Dexamethasone | □ | □ | □ | □ | □ |
| Combination medication #2: Prednisone | □ | □ | □ | □ | □ |
| Combination medication #2: Prednisolone | □ | □ | □ | □ | □ |
| Combination medication #2: Methylprednisolone | □ | □ | □ | □ | □ |
| Combination medication #3: Hydrocortisone | □ | □ | □ | □ | □ |
| Combination medication #3: Dexamethasone | □ | □ | □ | □ | □ |
| Combination medication #3: Prednisone | □ | □ | □ | □ | □ |
| Combination medication #3: Prednisolone | □ | □ | □ | □ | □ |
| Combination medication #3: Methylprednisolone | □ | □ | □ | □ | □ |

1. What androgen (or androgen precursor) lab values would you consider to be an indication of good control for your **adult** patients with classic CAH? *Please choose a range you tend to aim for with patients under your clinical care.* *Select one response per row*. ULN = upper limit of normal

| Androgen (or androgen precursor) | w/in  ULN | w/in  2X ULN | w/in  3X ULN | w/in  4x ULN | w/in  5x ULN | I do not have a lab range I treat to |
| --- | --- | --- | --- | --- | --- | --- |
| 17OH-Progesterone: |  |  |  |  |  |  |
| Males with TARTs | ⭘ | ⭘ | ⭘ | ⭘ | ⭘ | ⭘ |
| Males without TARTs | ⭘ | ⭘ | ⭘ | ⭘ | ⭘ | ⭘ |
| Females | ⭘ | ⭘ | ⭘ | ⭘ | ⭘ | ⭘ |
| A4 (androstenedione): |  |  |  |  |  |  |
| Males with TARTs | ⭘ | ⭘ | ⭘ | ⭘ | ⭘ | ⭘ |
| Males without TARTs | ⭘ | ⭘ | ⭘ | ⭘ | ⭘ | ⭘ |
| Females | ⭘ | ⭘ | ⭘ | ⭘ | ⭘ | ⭘ |
| Testosterone: |  |  |  |  |  |  |
| Females | ⭘ | ⭘ | ⭘ | ⭘ | ⭘ | ⭘ |

Optional: Please provide commentary for your above response.

1. Please indicate the hydrocortisone equivalency ratio you would find appropriate to use when summarizing a data set. Please answer with one number and not a range. Then, rank the certainty of the ratio chosen from 1-certain, low risk of being wrong to 5-unreliable, high risk of being wrong. *Note: This is NOT in reference to your clinical practice, but an opinion question (such as if reading a peer-reviewed article on a data from pharmacy claims, and the authors converted all the various glucocorticoid prescriptions and doses into hydrocortisone equivalents).*

| Glucocorticoid | Hydrocortisone dose equivalency ratio | 1-certain, low risk of being wrong  (±10% relative divergence possible) | 2-reliable, some risk of being wrong (±20%) | 3-less reliable, moderate risk of being wrong (±30%) | 4-risky, substantial risk of being wrong (±40%) | 5-unreliable, high risk of being wrong (more than 40%) |
| --- | --- | --- | --- | --- | --- | --- |
| Dexamethasone | ____ | ⭘ | ⭘ | ⭘ | ⭘ | ⭘ |
| Prednisone | ____ | ⭘ | ⭘ | ⭘ | ⭘ | ⭘ |
| Prednisolone | ____ | ⭘ | ⭘ | ⭘ | ⭘ | ⭘ |
| Methylprednisolone | ____ | ⭘ | ⭘ | ⭘ | ⭘ | ⭘ |
| Other: __________ | ____ | ⭘ | ⭘ | ⭘ | ⭘ | ⭘ |

Optional: Please provide any context/explanation for your ratios given above.

**NOW CONSIDER PHYSIOLOGIC GLUCOCORTICOID DOSES:** *Please answer the following questions based on your experience managing adult (18 years or older) patients with classic CAH.*

1. Physiologic replacement levels are often stated in ranges. What would you consider to be the upper end for a physiologic glucocorticoid dose with hydrocortisone? Note: Please state a number (no ranges).

_______mg/day

1. How certain do you feel this number represents the upper end of a physiologic glucocorticoid replacement dose?

- 1-certain, low risk of being wrong (±10% relative divergence possible)
- 2-reliable, some risk of being wrong (±20%)
- 3-risky, substantial risk of being wrong (±40%)
- 4-unreliable, high risk of being wrong (more than 40%)

Clinicians treating adult patients with classic CAH often need to balance supraphysiologic glucocorticoid treatment with its well-known adverse effects. Table 1 below illustrates four possible combinations of glucocorticoid dose treatment and the level of androgen and androgen-precursor control. Please review the table before answering the next set of questions.

**Table 1. Glucocorticoid Dose and Level of Androgen Control**

|  | Physiologic GC Dose | Supraphysiologic GC Dose |
| --- | --- | --- |
| Androgens NOT optimized | **Quadrant 1**  Physiologic GC doses  Androgens NOT optimized | **Quadrant 2**  Supraphysiologic GC doses  Androgens NOT optimized |
| Androgens optimized | **Quadrant 3**  Physiologic GC doses  Androgens optimized | **Quadrant 4**  Supraphysiologic GC doses  Androgens optimized |

*Please refer to Table 1 when answering the following questions.*

1. In your practice, what percentage of adult patients with classic CAH do you have in each of the four quadrants, at present? The sum should total to 100%.

Quadrant 1: ______ % patients

Quadrant 2: ______ % patients

Quadrant 3: ______ % patients

Quadrant 4: ______ % patients

Sum:

**Part 3. Exploring Unmet Needs for Adult Patients with CAH.** The following questions focus specifically on the unmet needs of your adult patients with classic CAH. Please respond based on your clinical experience:

1. What are the most important **short-term** unmet needs for your adult patients with classic CAH?
   ________________________________________
2. What are the most important **long-term** unmet needs for your adult patients with classic CAH?

________________________________________

1. What are the most important unmet needs specifically for your **female adult patients** with classic CAH? ________________________________________
2. What are the most important unmet needs specifically for your **male adult patients** with classic CAH?

________________________________________

1. What are the most important unmet needs for **younger adult patients** (18 to less than 55 years old, fertile ages/childbearing) with classic CAH? ________________________________________
2. What are the most important unmet needs for **older adult patients** (more than 55 years old, non-fertile, no longer childbearing) with classic CAH? ________________________________________
3. Please refer to Table 1 again.

|  | Physiologic GC Dose | Supraphysiologic GC Dose |
| --- | --- | --- |
| Androgens NOT optimized | **Quadrant 1**  Physiologic GC doses  Androgens NOT optimized | **Quadrant 2**  Supraphysiologic GC doses  Androgens NOT optimized |
| Androgens optimized | **Quadrant 3**  Physiologic GC doses  Androgens optimized | **Quadrant 4**  Supraphysiologic GC doses  Androgens optimized |

7.1 What are the most important unmet needs for patients in **Quadrant 1** (physiologic GC dose with androgens NOT optimized)? ________________________________________

7.2 What are the most important unmet needs for patients in **Quadrant 2** (supraphysiologic GC dose with androgens NOT optimized)?________________________________________

7.3 What are the most important unmet needs for patients in **Quadrant 3** (physiologic GC dose with androgens optimized)? ________________________________________

7.4 What are the most important unmet needs for patients in **Quadrant 4** (supraphysiologic GC dose with androgens optimized)? ________________________________________

1. Please rank the level of importance of the following attributes for adult patients with classic CAH due to their disease or to glucocorticoid-related complications.

| How important of a concern are each of the below disease or GC-related complications for adult patients with classic CAH? | Not at all important  1 | Somewhat important  2 | Moderately important  3 | Important  4 | Very important  5 |
| --- | --- | --- | --- | --- | --- |
| Cardiovascular and metabolic health |  |  |  |  |  |
| Pre-diabetes (IFG/IGT) | ⭘ | ⭘ | ⭘ | ⭘ | ⭘ |
| Type 2 diabetes | ⭘ | ⭘ | ⭘ | ⭘ | ⭘ |
| Hypertension | ⭘ | ⭘ | ⭘ | ⭘ | ⭘ |
| Overweight/obesity | ⭘ | ⭘ | ⭘ | ⭘ | ⭘ |
| Dyslipidemia (increased TG/low HDL) | ⭘ | ⭘ | ⭘ | ⭘ | ⭘ |
| Cardiovascular disease (for example coronary artery disease, stroke, etc.) | ⭘ | ⭘ | ⭘ | ⭘ | ⭘ |
| Other cardiovascular/metabolic concerns: ___________ | ⭘ | ⭘ | ⭘ | ⭘ | ⭘ |
| Bone health |  |  |  |  |  |
| Osteopenia/osteoporosis | ⭘ | ⭘ | ⭘ | ⭘ | ⭘ |
| Fragility fracture | ⭘ | ⭘ | ⭘ | ⭘ | ⭘ |
| Other bone health concerns: ___________ | ⭘ | ⭘ | ⭘ | ⭘ | ⭘ |
| Female health |  |  |  |  |  |
| Irregular menses/anovulation/amenorrhea | ⭘ | ⭘ | ⭘ | ⭘ | ⭘ |
| Infertility | ⭘ | ⭘ | ⭘ | ⭘ | ⭘ |
| Hirsutism/acne | ⭘ | ⭘ | ⭘ | ⭘ | ⭘ |
| Virilization | ⭘ | ⭘ | ⭘ | ⭘ | ⭘ |
| Other female health concerns: ___________ | ⭘ | ⭘ | ⭘ | ⭘ | ⭘ |
| Male health |  |  |  |  |  |
| Testicular adrenal rest tumors (TARTs) | ⭘ | ⭘ | ⭘ | ⭘ | ⭘ |
| Infertility | ⭘ | ⭘ | ⭘ | ⭘ | ⭘ |
| Other male health concerns: ___________ | ⭘ | ⭘ | ⭘ | ⭘ | ⭘ |
| Psychosocial health and well-being |  |  |  |  |  |
| Anxiety | ⭘ | ⭘ | ⭘ | ⭘ | ⭘ |
| Depression | ⭘ | ⭘ | ⭘ | ⭘ | ⭘ |
| Decreased sexual satisfaction | ⭘ | ⭘ | ⭘ | ⭘ | ⭘ |
| Reduced vitality | ⭘ | ⭘ | ⭘ | ⭘ | ⭘ |
| Impaired cognition | ⭘ | ⭘ | ⭘ | ⭘ | ⭘ |
| Insomnia, poor sleep quality | ⭘ | ⭘ | ⭘ | ⭘ | ⭘ |
| Fatigue | ⭘ | ⭘ | ⭘ | ⭘ | ⭘ |
| Other psychosocial health and well-being concerns: ___________ | ⭘ | ⭘ | ⭘ | ⭘ | ⭘ |
| Other complications |  |  |  |  |  |
| Increased infections | ⭘ | ⭘ | ⭘ | ⭘ | ⭘ |
| Skin bruising, thinning, fragility | ⭘ | ⭘ | ⭘ | ⭘ | ⭘ |
| Myopathy | ⭘ | ⭘ | ⭘ | ⭘ | ⭘ |
| Ocular (ex: glaucoma, cataracts) | ⭘ | ⭘ | ⭘ | ⭘ | ⭘ |
| Other complications/concerns: ___________ | ⭘ | ⭘ | ⭘ | ⭘ | ⭘ |

**Part 4. Changes in glucocorticoid treatment regimen.** The following questions focus specifically on glucocorticoid treatment of adult patients with classic CAH. Please respond based on your clinical experience. Please note, change is defined as any:

- Change in type of glucocorticoid
- Change in dose (mg)
- Change does NOT include changes for stress or sick day dosing

1. In your opinion, how often does the **average adult patient** with classic CAH change their usual glucocorticoid regimen?

- Once per month
- Once every 2 months
- Once per quarter
- 3 times per year
- Twice yearly
- Once yearly
- Every other year
- Every 3-5 years
- Less frequently than every 5 years

1. What are some of the factors that contribute to patients with CAH changing their glucocorticoid regimen? ________________________________________

**Please refer to Table 1 again.**

|  | Physiologic GC Dose | Supraphysiologic GC Dose |
| --- | --- | --- |
| Androgens NOT optimized | **Quadrant 1**  Physiologic GC doses  Androgens NOT optimized | **Quadrant 2**  Supraphysiologic GC doses  Androgens NOT optimized |
| Androgens optimized | **Quadrant 3**  Physiologic GC doses  Androgens optimized | **Quadrant 4**  Supraphysiologic GC doses  Androgens optimized |

1. In your opinion, how often does the average patient in **Quadrant 1 (physiologic GC dose with androgens NOT optimized)** change their glucocorticoid regimen?

- Once per month
- Once every 2 months
- Once per quarter
- 3 times per year
- Twice yearly
- Once yearly
- Every other year
- Every 3-5 years
- Less frequently than every 5 years

1. In your opinion, how often does the average patient in **Quadrant 2 (supraphysiologic GC dose with androgens NOT optimized)** change their glucocorticoids?

- Once per month
- Once every 2 months
- Once per quarter
- 3 times per year
- Twice yearly
- Once yearly
- Every other year
- Every 3-5 years
- Less frequently than every 5 years

1. In your opinion, how often does the average patient in **Quadrant 3 (physiologic GC dose with androgens optimized)** change their glucocorticoids?

- Once per month
- Once every 2 months
- Once per quarter
- 3 times per year
- Twice yearly
- Once yearly
- Every other year
- Every 3-5 years
- Less frequently than every 5 years

1. In your opinion, how often does the average patient in **Quadrant 4 (supraphysiologic GC dose with androgens optimized)** change their glucocorticoids?

- Once per month
- Once every 2 months
- Once per quarter
- 3 times per year
- Twice yearly
- Once yearly
- Every other year
- Every 3-5 years
- Less frequently than every 5 years

**Round 2 Delphi Questionnaire**

**Part 1. GC Management.** *Please answer the following questions based on managing adults (18 years or older) with classic CAH.*

1. The following table displays a summary of survey round 1 results for the percentage of **adult** patients with classic CAH treated with various GC regimens. Please review and take into consideration the summary results before responding to the question. **Do you agree with the repartition below indicating hydrocortisone as the most widely used GC globally?**

□ Yes—agree

□ No—disagree

| Round 1: What Percentage of Adult Patients with Classic CAH Are on each of the Following GC Regimens at Present?, % | Your Response from Round 1 | Mean (SD) | Median [Range] |
| --- | --- | --- | --- |
| Hydrocortisone only |  | 62.1 (32.6) | 65.0 [10.0–96.0] |
| Dexamethasone only |  | 5.6 (6.7) | 3.0 [0.0–20.0] |
| Prednisone only |  | 14.4 (26.4) | 5.0 [0.0–80.0] |
| Prednisolone only |  | 10.6 (26.3) | 0.0 [0.0–80.0] |
| Methylprednisolone only |  | 2.8 (8.3) | 0.0 [0.0–25.0] |
| Combination GC (e.g., hydrocortisone and dexamethasone; hydrocortisone and prednisone; hydrocortisone and prednisolone; hydrocortisone and methylprednisolone) |  | 17.6 (33.6) | 5.0 [1.0–100.0] |

Abbreviations: CAH = congenital adrenal hyperplasia; GC = glucocorticoid

1. The following table displays a summary of survey round 1 results for the total daily dose prescribed for various GC regimens for **adult** patients with classic CAH. Please review and take into consideration the summary results before responding to the question.

| Round 1: What is the Total Daily Dose You Typically Use for the Treatment of Adult Patients with Classic CAH for each of the GC Regimens Below? mg/day | Your Response from Round 1 | Clinicians Reporting a Single Number  Round 1 Results | | Clinicians Reporting a Range  Round 1 Results | | Round 2: What Do You Think Is the Average Dose of each Medication or Combination Used Today, Globally? mg/day |
| --- | --- | --- | --- | --- | --- | --- |
|  | Number and/or Range | Mean (SD) | Median [Range] | Lower Range  Mean (SD) | Upper Range  Mean (SD) | **(Enter a NUMBER or RANGE)** |
| Hydrocortisone |  | 26.3 (4.8) | 27.5 [20.0–30.0] | 15.6 (4.2) | 40.6 (9.4) |  |
| Dexamethasone |  | 0.6 (0.5) | 0.6 [0.3–1.0] | 0.5 (0.1) | 1.6 (0.5) |  |
| Prednisone |  | 4.0 (1.4) | 4.0 [3.0–5.0] | 3.8 (1.8) | 6.2 (2.1) |  |
| Prednisolone |  | 5.0 (---) | 5.0 [5.0–5.0] | 2.8 (2.0) | 5.8 (1.4) |  |
| Methylprednisolone |  | 6.0 (---) | 6.0 [6.0–6.0] | 4.0 (---) | 8.0 (---) |  |

Abbreviations: CAH = congenital adrenal hyperplasia; GC = glucocorticoid

1. This screen displays a summary of survey round 1 results for determining what androgen (or androgen precursor) lab values indicate good control for your **adult** patients with classic CAH. Please review and take into consideration the summary results before responding to the questions. *Summary results from round 1 are in italics. Your response from round 1 is highlighted BLUE, and the response selected by most participants is highlighted in GREEN where there is a clear majority.*
2. What lab values for **17OH progesterone** are considered to be an indication of good control for **adult** patients with classic CAH?

| Androgen (or androgen precursor) | Within ULN | Within  2X ULN | Within  3X ULN | Within  4X ULN | I Do Not Have a Lab Range I Treat to |
| --- | --- | --- | --- | --- | --- |
| 17OH-Progesterone |  |  |  |  |  |
| Males with TARTs | 🞎 | 🞎 | 🞎 | 🞎 | 🞎 |
| *Round 1 results* | *11%* | ***44%*** | *11%* | *0%* | *33%* |
| Males without TARTs | 🞎 | 🞎 | 🞎 | 🞎 | **🞎** |
| *Round 1 results* | *0%* | *11%* | *22%* | *22%* | ***45%*** |
| Females | 🞎 | 🞎 | 🞎 | 🞎 | 🞎 |
| *Round 1 results* | *0%* | *22%* | ***45%*** | *0%* | *33%* |

Abbreviations: TART = testicular adrenal rest tumor; ULN = upper limit of normal

1. What lab values for **A4 (androstenedione)** are considered to be an indication of good control for **adult** patients with classic CAH?

| Androgen (or androgen precursor) | Within ULN | Within  2X ULN | Within  3X ULN | Within  4X ULN | I Do Not Have a Lab Range I Treat to |
| --- | --- | --- | --- | --- | --- |
| A4 (Androstenedione) |  |  |  |  |  |
| Males with TARTs | **🞎** | 🞎 | 🞎 | 🞎 | 🞎 |
| *Round 1 results* | **56%** | 22% | 11% | 0% | 11% |
| Males without TARTs | 🞎 | 🞎 | 🞎 | 🞎 | 🞎 |
| *Round 1 results* | 22% | 22% | 11% | 22% | 22% |
| Females | 🞎 | 🞎 | 🞎 | 🞎 | 🞎 |
| *Round 1 results* | 33% | 22% | 33% | 0% | 11% |

Abbreviations: TART = testicular adrenal rest tumor; ULN = upper limit of normal

1. What lab values for **testosterone** are considered to be an indication of good control for **adult** patients with classic CAH?

| Androgen (or androgen precursor) | Within ULN | Within  2X ULN | Within  3X ULN | Within  4X ULN | I Do Not Have a Lab Range I Treat to |
| --- | --- | --- | --- | --- | --- |
| Testosterone | | | | | |
| Females | **🞎** | 🞎 | 🞎 | 🞎 | 🞎 |
| *Round 1 results* | **56%** | 22% | 11% | 0% | 11% |

Abbreviation: ULN = upper limit of normal

D. Which of the following is the leading factor for treatment decisions related to labs? *Select one response.*

□ Lab values (17-OHP, A4, and testosterone) are the leading indicators of control.

□ Clinical presentation is the leading indicator of control.

□ The balance of clinical presentation and lab values is the leading indicator of control.

1. The table below displays a summary of survey round 1 results for typical timing for GC administration in **adult** patients with classic CAH. Please review and take into consideration the summary results before responding to the questions. In round 1:

- **100%** of clinicians reported dosing timing for hydrocortisone in the morning and **89%** reported prescribing the dose again in the afternoon.
- Dexamethasone was mostly prescribed at bedtime (**83%**).

| Glucocorticoid | Morning | Afternoon | Evening | Bedtime |
| --- | --- | --- | --- | --- |
| Hydrocortisone | **100%** | **89%** | 78% | 22% |
| Dexamethasone | 17% | - | 17% | **83%** |
| Prednisone | 80% | 20% |  | 60% |
| Prednisolone | 100% | 33% | 33% | 33% |
| Methylprednisolone | 100% | - | - | 100% |

1. Do you agree that dexamethasone should be prescribed at bedtime, if given once daily?

□ Yes—agree

□ No—disagree

A total of 22% of respondents noted that the timing of when an androgen lab test is performed in relation to patient’s GC administration will have an important impact on what lab values can be considered an indicator of good control.

1. What is the **optimal** timing for lab testing of androgens in **adults** with classic CAH?

□ Before morning GC dose

□ After morning GC dose

□ Before afternoon GC dose

□ After afternoon GC dose

□ Closer to evening or bedtime GC dose

1. In round 1, you were asked to provide the hydrocortisone equivalency ratio they found appropriate to use when summarizing a data set. *Below is a summary of the mean equivalency ratio for GCs. Please review each result from round 1 and confirm your agreement with the findings.*

| Glucocorticoid | Hydrocortisone Dose Equivalency Ratio  Mean (SD), Range | Example Conversion | I Agree with the Round 1 Results for the Hydrocortisone Dose Equivalency Ratio | | If you selected No, please provide a value that you consider to be appropriate. A*nswer with* *number and not a range.* |
| --- | --- | --- | --- | --- | --- |
|  |  |  | **Yes** | **No** |  |
| Dexamethasone | 43.1 (25.4), [25.0–80.0]  [rounded to 45] | **0.375 mg** of dexamethasone is equivalent to 17 mg hydrocortisone  (e.g., 0.375 mg × 45 = 17) | 🞎 | 🞎 | _____ mg/day |
| Prednisone | 4.4 (0.5), [4.0–5.0] | **7 mg** of prednisone is equivalent to 28–35 mg of hydrocortisone  (e.g., 7 mg × 4 = 28,  7 mg × 5 = 35) | 🞎 | 🞎 | _____ mg/day |
| Prednisolone | 4.4 (0.5), [4.0–5.0] | **5 mg** of prednisone is equivalent to 20–25 mg of hydrocortisone  (e.g., 5 mg × 4 = 20,  5 mg × 5 = 25) | 🞎 | 🞎 | _____ mg/day |
| Methylprednisolone | 4.8 (0.4), [4.0–5.0] | **10 mg** of prednisone is equivalent to 40–50 mg of hydrocortisone  (e.g., 10 mg × 4 = 40,  10 mg × 5 = 50) | 🞎 | 🞎 | _____ mg/day |

Abbreviation: SD = standard deviation

1. Considering a physiologic dose **provides approximately the same GC effects as normal cortisol production**, indicate what would you consider to be the **upper end** for a physiologic GC dose with hydrocortisone? *Please answer with one number and not a range. Please note that the units are mg/day and not mg/m^2^/day.*

- Round 1 summary results: 27.2 (9.7), [15–40] mg
- Your Round 1 response: _______ mg

Do you agree that 25 – 30 mg is the upper end for a physiologic GC dose of hydrocortisone?

□ Yes

□ No

What would be more appropriate?

_____ mg/day

B. How certain do you feel your round 2 response represents the upper end of a physiologic GC replacement dose?

🞎 1: Certain, low risk of being wrong (±10% relative divergence possible)

🞎 2: Reliable, some risk of being wrong (±20%)

🞎 3: Risky, substantial risk of being wrong (±40%)

🞎 4: Unreliable, great risk of being wrong (>40%)

**Part 2. Exploring Unmet Needs for Adults with CAH.** *Please answer the following questions based on managing adults (18 years or older) with classic CAH.* The following questions focus specifically on the unmet needs of your adult patients with classic CAH. Please respond based on your clinical experience:

In round 1, physicians were asked to describe the most important unmet needs for adults with classic CAH, based on key subgroups of interest:

- Short- and long-term unmet needs
- Females and males
- Younger (18 to ≤55 years old) and older (>55 years old) patients
- Quadrants one, two, three, and four (Table 1)

|  | Physiologic GC Dose | Supraphysiologic GC Dose |
| --- | --- | --- |
| Androgens NOT optimized | **Quadrant 1**  Physiologic GC doses  Androgens NOT optimized | **Quadrant 2**  Supraphysiologic GC doses  Androgens NOT optimized |
| Androgens optimized | **Quadrant 3**  Physiologic GC doses  Androgens optimized | **Quadrant 4**  Supraphysiologic GC doses  Androgens optimized |

Based on the open-ended responses from round 1, the study team developed high-level categories to describe the responses. The three categories of unmet needs for adults with classic CAH identified were:

- Good androgen control/avoidance of hyperandrogenism (including improving fertility, regaining regular periods, exploring androgen replacement therapy, and reducing hirsutism)
- Managing/reducing supraphysiologic GC doses (including preventing consequences related to cardiovascular health, bone health, and metabolic health)
- Treatment-related needs (including simplified dosing to enhance compliance and an affordable option) and the need for a new treatment (such as hormone replacement or mineralocorticoid therapies that control androgens without side effects)

The relative importance of these categories of unmet needs varied across patient groups and key findings are summarized in the questions below for further exploration and confirmation.

1. Over half of respondents (56%) noted an unmet need for new treatments across all adult CAH subgroups in round 1, most commonly noted as a short-term need. Would you agree that there is an important unmet need for **new treatments across all adult CAH subgroups**?

- Yes
- No

1. How important is each of the following unmet needs in the **short-term** for adults with CAH? *The proportion of clinicians who mentioned this unmet need in round 1 are presented in the second column.*

| Unmet Need | Round 1 Summary (% reporting unmet need) | 1  Not at All Important | 2  Somewhat Important | 3 Moderately Important | 4  Important | 5  Very Important |
| --- | --- | --- | --- | --- | --- | --- |
| Good androgen control/ hyperandrogenism (including fertility, ARTS, and hirsutism) | *56%* | 🞎 | 🞎 | 🞎 | 🞎 | 🞎 |
| Managing/reducing supraphysiologic GC doses (including consequences related to CV health, bone health, and metabolic health) | *56%* | 🞎 | 🞎 | 🞎 | 🞎 | 🞎 |
| Treatment-related needs and need for a new treatment (including needs of simplified dosing and affordability) | *44%* | 🞎 | 🞎 | 🞎 | 🞎 | 🞎 |

Abbreviations: ART = androgen replacement therapy; CV = cardiovascular; GC = glucocorticoid

1. How important is each of the following unmet needs in the **long-term** for adults with CAH? *The proportion of clinicians who mentioned this unmet need in round 1 are presented in the second column.*

| Unmet Need | Round 1 Summary (% reporting unmet need) | 1  Not at All Important | 2  Somewhat Important | 3 Moderately Important | 4  Important | 5  Very Important |
| --- | --- | --- | --- | --- | --- | --- |
| Good androgen control/ hyperandrogenism (including fertility, ARTS, and hirsutism) | *56%* | 🞎 | 🞎 | 🞎 | 🞎 | 🞎 |
| Managing/reducing supraphysiologic GC doses (including consequences related to CV health, bone health, and metabolic health) | *67%* | 🞎 | 🞎 | 🞎 | 🞎 | 🞎 |
| Treatment-related needs and need for a new treatment (including needs of simplified dosing and affordability) | *22%* | 🞎 | 🞎 | 🞎 | 🞎 | 🞎 |

Abbreviations: ART = androgen replacement therapy; CV = cardiovascular; GC = glucocorticoid

1. How important is each of the following unmet needs for **female** adults with CAH? *The proportion of clinicians who mentioned this unmet need in round 1 are presented in the second column.*

| Unmet Need | Round 1 Summary (% reporting unmet need) | 1  Not at All Important | 2  Somewhat Important | 3 Moderately Important | 4  Important | 5  Very Important |
| --- | --- | --- | --- | --- | --- | --- |
| Good androgen control/ hyperandrogenism (including fertility, ARTS, and hirsutism) | *89%* | 🞎 | 🞎 | 🞎 | 🞎 | 🞎 |
| Managing/reducing supraphysiologic GC doses (including consequences related to CV health, bone health, and metabolic health) | *22%* | 🞎 | 🞎 | 🞎 | 🞎 | 🞎 |
| Treatment-related needs and need for a new treatment (including needs of simplified dosing and affordability) | *11%* | 🞎 | 🞎 | 🞎 | 🞎 | 🞎 |

Abbreviations: ART = androgen replacement therapy; CV = cardiovascular; GC = glucocorticoid

1. How important is each of the following unmet needs for **male** adults with CAH? *The proportion of clinicians who mentioned this unmet need in round 1 are presented in the second column.*

| Unmet Need | Round 1 Summary (% reporting unmet need) | 1  Not at All Important | 2  Somewhat Important | 3 Moderately Important | 4  Important | 5  Very Important |
| --- | --- | --- | --- | --- | --- | --- |
| Good androgen control/ hyperandrogenism (including fertility, ARTS, and hirsutism) | *89%* | 🞎 | 🞎 | 🞎 | 🞎 | 🞎 |
| Managing/reducing supraphysiologic GC doses (including consequences related to CV health, bone health, and metabolic health) | *22%* | 🞎 | 🞎 | 🞎 | 🞎 | 🞎 |
| Treatment-related needs and need for a new treatment (including needs of simplified dosing and affordability) | *11%* | 🞎 | 🞎 | 🞎 | 🞎 | 🞎 |

Abbreviations: ART = androgen replacement therapy; CV = cardiovascular; GC = glucocorticoid

1. How important is each of the following unmet needs for **younger adult (18 to ≤55 years old)** patients with CAH? *The proportion of clinicians who mentioned this unmet need in round 1 are presented in the second column.*

| Unmet Need | Round 1 Summary (% reporting unmet need) | 1  Not at All Important | 2  Somewhat Important | 3 Moderately Important | 4  Important | 5  Very Important |
| --- | --- | --- | --- | --- | --- | --- |
| Good androgen control/ hyperandrogenism (including fertility, ARTS, and hirsutism) | *78%* | 🞎 | 🞎 | 🞎 | 🞎 | 🞎 |
| Managing/reducing supraphysiologic GC doses (including consequences related to CV health, bone health, and metabolic health) | *56%* | 🞎 | 🞎 | 🞎 | 🞎 | 🞎 |
| Treatment-related needs and need for a new treatment (including needs of simplified dosing and affordability) | *11%* | 🞎 | 🞎 | 🞎 | 🞎 | 🞎 |

Abbreviations: ART = androgen replacement therapy; CV = cardiovascular; GC = glucocorticoid

1. How important is each of the following unmet needs for **older (>55 years old)** patients with CAH? *The proportion of clinicians who mentioned this unmet need in round 1 are presented in the second column.*

| Unmet Need | Round 1 Summary (% reporting unmet need) | 1  Not at All Important | 2  Somewhat Important | 3 Moderately Important | 4  Important | 5  Very Important |
| --- | --- | --- | --- | --- | --- | --- |
| Managing/reducing supraphysiologic GC doses (including consequences related to CV health, bone health, and metabolic health) | *78%* | 🞎 | 🞎 | 🞎 | 🞎 | 🞎 |
| Other (i.e. adrenal enlargement) | *11%* | 🞎 | 🞎 | 🞎 | 🞎 | 🞎 |

Abbreviations: CV = cardiovascular; GC = glucocorticoid

Clinicians treating adults with classic CAH often need to balance supraphysiologic GC treatment with its well-known adverse effects. Table 1 illustrates four possible combinations of GC dose treatment and the level of androgen and androgen-precursor control. Please review the table before answering the next set of questions.

Table 1. GC Dose and Level of Androgen Control

|  | Physiologic GC Dose | Supraphysiologic GC Dose |
| --- | --- | --- |
| Androgens NOT optimized | **Quadrant 1**  Physiologic GC doses  Androgens NOT optimized | **Quadrant 2**  Supraphysiologic GC doses  Androgens NOT optimized |
| Androgens optimized | **Quadrant 3**  Physiologic GC doses  Androgens optimized | **Quadrant 4**  Supraphysiologic GC doses  Androgens optimized |

Abbreviation: GC = glucocorticoid

*Please refer to Table 1 when answering the following questions.*

1. How important is each of the following unmet needs for patients with CAH in **quadrant 1 (physiologic GC doses and androgens NOT optimized)**? *The proportion of clinicians who mentioned this unmet need in round 1 are presented in the second column.*

| Unmet Need | Round 1 Summary (% reporting unmet need) | 1  Not at All Important | 2  Somewhat Important | 3 Moderately Important | 4  Important | 5  Very Important |
| --- | --- | --- | --- | --- | --- | --- |
| Good androgen control/ hyperandrogenism (including fertility, ARTS, and hirsutism) | *89%* | 🞎 | 🞎 | 🞎 | 🞎 | 🞎 |
| Other (i.e., adrenal enlargement) | *11%* | 🞎 | 🞎 | 🞎 | 🞎 | 🞎 |

Abbreviation: ART = androgen replacement therapy

1. How important is each of the following unmet needs for patients with CAH in **quadrant 2 (supraphysiologic GC doses and androgens NOT optimized)**? *The proportion of clinicians who mentioned this unmet need in round 1 are presented in the second column.*

| Unmet Need | Round 1 Summary (% reporting unmet need) | 1  Not at All Important | 2  Somewhat Important | 3 Moderately Important | 4  Important | 5  Very Important |
| --- | --- | --- | --- | --- | --- | --- |
| Good androgen control/ hyperandrogenism (including fertility, ARTS, and hirsutism) | *56%* | 🞎 | 🞎 | 🞎 | 🞎 | 🞎 |
| Managing/reducing supraphysiologic GC doses (including consequences related to CV health, bone health, and metabolic health) | *67%* | 🞎 | 🞎 | 🞎 | 🞎 | 🞎 |
| Treatment-related needs and need for a new treatment (including needs of simplified dosing and affordability) | *11%* | 🞎 | 🞎 | 🞎 | 🞎 | 🞎 |

Abbreviations: ART = androgen replacement therapy; CV = cardiovascular; GC = glucocorticoid

1. How important is each of the following unmet needs for patients with CAH in **quadrant 3 (physiologic GC doses and androgens optimized)**? *The proportion of clinicians who mentioned this unmet need in round 1 are presented in the second column.*

| Unmet Need | Round 1 Summary (% reporting unmet need) | 1  Not at All Important | 2  Somewhat Important | 3 Moderately Important | 4  Important | 5  Very Important |
| --- | --- | --- | --- | --- | --- | --- |
| Managing/reducing supraphysiologic GC doses (including consequences related to CV health, bone health, and metabolic health) | *11%* | 🞎 | 🞎 | 🞎 | 🞎 | 🞎 |
| Treatment-related needs and need for a new treatment (including needs of simplified dosing and affordability) | *22%* | 🞎 | 🞎 | 🞎 | 🞎 | 🞎 |
| There is no unmet need for patients in quadrant 3 | *33%* | 🞎 | 🞎 | 🞎 | 🞎 | 🞎 |
| Other (i.e., adrenal crisis, psychological support, long-term maintenance) | *33%* | 🞎 | 🞎 | 🞎 | 🞎 | 🞎 |

Abbreviations: CV = cardiovascular; GC = glucocorticoid

1. How important is each of the following unmet needs for patients with CAH in **quadrant 4 (supraphysiologic GC doses and androgens optimized)**? *The proportion of clinicians who mentioned this unmet need in round 1 are presented in the first column.*

| Unmet Need | Round 1 Summary (% reporting unmet need) | 1  Not at All Important | 2  Somewhat Important | 3 Moderately Important | 4  Important | 5  Very Important |
| --- | --- | --- | --- | --- | --- | --- |
| Good androgen control/ hyperandrogenism (including fertility, ARTS, and hirsutism) | *11%* | 🞎 | 🞎 | 🞎 | 🞎 | 🞎 |
| Managing/reducing supraphysiologic GC doses (including consequences related to CV health, bone health, and metabolic health) | *89%* | 🞎 | 🞎 | 🞎 | 🞎 | 🞎 |
| Treatment-related needs and need for a new treatment (including needs of simplified dosing and affordability) | *11%* | 🞎 | 🞎 | 🞎 | 🞎 | 🞎 |

Abbreviations: ART = androgen replacement therapy; CV = cardiovascular; GC = glucocorticoid

In round 1, clinicians were asked to rank the level of importance of differing attributes for adults with classic CAH due to their disease or GC-related complications. The attributes included:

- Cardiovascular and metabolic health
- Bone health
- Female health
- Male health
- Psychosocial health and well-being
- Other complications

The next set of questions will provide your individual response from round 1 in the **first** column and a summary of the results in the **second** column. Please review the results and indicate your response for round 2.Round 1 response options were:

| How important of a concern are each of the below disease or GC-related complications for adult patients with classic CAH? | 1 Not at all important | 2 Somewhat important | 3 Moderately important | 4  Important | 5  Very important |
| --- | --- | --- | --- | --- | --- |

1. For round 2, please indicate whether you agree that each of the concerns related to **cardiovascular and metabolic health** listed in the table below are **important or very important** on a 5-point scale ranging from “not at all important” to “very important.”

| How important of a concern are each of the below disease or GC-related complications for adults with classic CAH? | Your Response from Round 1 | Round 1  Mean [Range] | Do You Agree that the Complication Is Important or Very Important (4–5)? | |
| --- | --- | --- | --- | --- |
| CV and metabolic health |  |  | **Yes** | **No** |
| Pre-diabetes (IFG/IGT) |  | *4 [2 –5]* | 🞎 | 🞎 |
| Type 2 diabetes |  | *4 [2–5]* | 🞎 | 🞎 |
| Hypertension |  | *4 [2–5]* | 🞎 | 🞎 |
| Overweight/obesity |  | *4.6 [4–5]* | 🞎 | 🞎 |
| CV disease (e.g., coronary artery disease, stroke) |  | *4.4 [2–5]* | 🞎 | 🞎 |
| Other: change in body composition |  | *4 [4–4]* | 🞎 | 🞎 |

Abbreviations: CAH = congenital adrenal hyperplasia; CV = cardiovascular; GC = glucocorticoid; IFG = impaired fasting glucose;
IGT = impaired glucose tolerance

1. For round 2, please indicate whether you agree that the concern related to **cardiovascular and metabolic health** listed in the table below are **moderately important** on a 5-point scale ranging from “not at all important” to “very important.”

| How important of a concern are each of the below disease or GC-related complications for adults with classic CAH? | Your Response from Round 1 | Round 1  Mean [Range] | Do You Agree that the Complication Is Moderately Important (3)? | |
| --- | --- | --- | --- | --- |
| CV and metabolic health |  |  | **Yes** | **No** |
| Dyslipidemia (increased TG/low HDL) |  | *3.9 [2 –5]* | 🞎 | 🞎 |

Abbreviations: CAH = congenital adrenal hyperplasia; CV = cardiovascular; GC = glucocorticoid; HDL = high-density lipoprotein;
TG = triglycerides

1. For round 2, please indicate whether you agree that each of the concerns related to **bone health** listed in the table below are **important or very important** on a 5-point scale ranging from “not at all important” to “very important.”

| How important of a concern are each of the below disease or GC-related complications for adults with classic CAH? | Your Response from Round 1 | Round 1  Mean [Range] | Do You Agree that the Complication Is Important or Very Important (4–5)? | |
| --- | --- | --- | --- | --- |
| Bone health |  |  | Yes | No |
| Osteopenia/osteoporosis |  | *4.3 [3–5]* | 🞎 | 🞎 |
| Fragility fracture |  | *4.5 [3–5]* | 🞎 | 🞎 |

Abbreviations: CAH = congenital adrenal hyperplasia; GC = glucocorticoid

1. For round 2, please indicate whether you agree that each of the concerns related to **female and male health** listed in the table below are **important or very important** on a 5-point scale ranging from “not at all important” to “very important.”

| How important of a concern are each of the below disease or GC-related complications for adults with classic CAH? | Your Response from Round 1 | Round 1  Mean [Range] | Do You Agree that the Complication Is Important or Very Important (4–5)? | |
| --- | --- | --- | --- | --- |
| Female health |  |  | Yes | No |
| Irregular menses/anovulation/amenorrhea |  | *4.1 [3–5]* | 🞎 | 🞎 |
| Hirsutism/acne |  | *4.1 [3–5]* | 🞎 | 🞎 |
| Virilization |  | *4.3 [1–5]* | 🞎 | 🞎 |
| Male health |  |  |  |  |
| TARTs |  | *4.7 [2–5]* | 🞎 | 🞎 |

Abbreviations: CAH = congenital adrenal hyperplasia; GC = glucocorticoid; TART = testicular adrenal rest tumor

1. For round 2, please indicate whether you agree that each of the concerns related to **psychosocial health and well-being** listed in the table below are **important or very important** on a 5-point scale ranging from “not at all important” to “very important.”

| How important of a concern are each of the below disease or GC-related complications for adults with classic CAH? | Your Response from Round 1 | Round 1  Mean [Range] | Do You Agree that the Complication Is Important or Very Important (4–5)? | |
| --- | --- | --- | --- | --- |
| Psychosocial health and well-being |  |  | **Yes** | **No** |
| Depression |  | *4.11 [2–5]* | 🞎 | 🞎 |
| Decreased sexual satisfaction |  | *4 [2–5]* | 🞎 | 🞎 |

Abbreviations: CAH = congenital adrenal hyperplasia; GC = glucocorticoid

1. For round 2, please indicate whether you agree that the concern related to **psychosocial health and well-being** listed in the table below are **moderately important** on a 5-point scale ranging from “not at all important” to “very important.”

| How important of a concern are each of the below disease or GC-related complications for adults with classic CAH? | Your Response from Round 1 | Round 1  Mean [Range] | Do You Agree that the Complication Is Moderately Important (3)? | |
| --- | --- | --- | --- | --- |
| Psychosocial health and well-being |  |  | **Yes** | **No** |
| Anxiety |  | *3.89 [2–5]* | 🞎 | 🞎 |
| Reduced vitality |  | *3.78 [3–5]* | 🞎 | 🞎 |
| Impaired cognition |  | *3.56 [2–5]* | 🞎 | 🞎 |
| Insomnia, poor sleep quality |  | *3.56 [2–5]* | 🞎 | 🞎 |
| Fatigue |  | *3.67 [2–5]* | 🞎 | 🞎 |

Abbreviations: CAH = congenital adrenal hyperplasia; GC = glucocorticoid

1. For round 2, please indicate whether you agree that each of the concerns related to **other complications** listed in the table below are **moderately** **important** on a 5-point scale ranging from “not at all important” to “very important.”

| How important of a concern are each of the below disease or GC-related complications for adults with classic CAH? | Your Response from Round 1 | Round 1  Mean [Range] | Do You Agree that the Complication Is Moderately Important (3)? | |
| --- | --- | --- | --- | --- |
| Other Complications |  |  | **Yes** | **No** |
| Increased infections |  | *3.6 [2–5]* | 🞎 | 🞎 |
| Skin bruising, thinning, fragility |  | *3.8 [2–5]* | 🞎 | 🞎 |
| Myopathy |  | *3.9 [2–5]* | 🞎 | 🞎 |
| Ocular (ex: glaucoma, cataracts) |  | *3.6 [2–5]* | 🞎 | 🞎 |

Abbreviations: CAH = congenital adrenal hyperplasia; GC = glucocorticoid
